# Meeting abstracts of the GISM Annual Meeting 2025

**DOI:** 10.20517/evcna.2025.53

**Published:** 2025-08-04

**Authors:** Enrico Ragni, Enrico Lucarelli, Stefano Grolli, Antonietta Rosa Silini, Valentina Grespi, Ivana Ferrero, Luisa Pascucci, Michela Pozzobon

**Affiliations:** ^1^Laboratorio di Biotecnologie Applicate all’Ortopedia, IRCCS Ospedale Galeazzi-Sant’Ambrogio, Milano 20157, Italy.; ^2^Osteoncology, Bone and Soft Tissue Sarcomas and Innovative Therapies Unit, IRCCS Istituto Ortopedico Rizzoli, Bologna 40136, Italy.; ^3^Department of Veterinary Medical Science, University of Parma, Parma 43121, Italy.; ^4^Centro di Ricerca E. Menni, Fondazione Poliambulanza Istituto Ospedaliero, Brescia 25124, Italy.; ^5^Laboratorio Cellule Staminali, Cell Factory e Biobanca, AOSP Santa Maria, Terni 05100, Italy.; ^6^Stem Cell Transplantation and Cellular Therapy Laboratory, Paediatric Onco-Haematology Division, Regina Margherita Children’s Hospital, City of Health and Science of Turin, Torino 10126, Italy.; ^7^Department of Veterinary Medicine, University of Perugia, Perugia 06126, Italy.; ^8^Department of Women’s and Children’s Health, University of Padua, Padova 35128, Italy.; ^9^Foundation Institute of Pediatric Research Città della Speranza, Padova 35127, Italy.

The GISM Annual Meeting 2025, May 8-9, 2025 [Table 1].

**Table 1:** Table of contents

**No.**	**Abstract title**	**Authors**	**Page**
1	Impact of platelet-rich plasma on gene expression in bovine endometrial mesenchymal stem cells infected with bovine gammaherpesvirus type 4 and exposed to lipopolysaccharide	Valentina Andreoli, Sofia Lopez, Santiago Delgado, Sandra Perez, Susana Pereyra, Florencia Romeo, Stefano Grolli, Andrea Elizabeth Verna	4
2	Case report: Treatment of ununited anconeal process (UAP) with microfragmented fat in a dog	Alessandro Guglielmo Aspesi, Maurizio Del Bue, Priscilla Berni, Stefano Grolli	5
3	Extracellular vesicles isolated from mesenchymal stromal cells loaded with doxorubicin: Proof of concept for a new osteosarcoma treatment	Alessia Santa Giovanna Banche Niclot, Camilla Francesca Proto, Federico Divincenzo, Francesco Barbero, Ivana Fenoglio, Chiara Scarpa, Alessandro Barge, Francesca Paino, Ivana Ferrero, Elisa Tirtei, Katia Mareschi, Franca Fagioli	5
4	Freeze-dried mesenchymal stem cell secretome for osteoarthritis regenerative medicine: Clinical evaluation in dogs and horses	Elia Bari, Priscilla Berni, Maurizio Del Bue, Virna Conti, Valentina Andreoli, Roberto Ramoni, Mario Angelone, Gian Paolo Squassino, Maurizio Rinaldi, Silvia Dotti, Rossana Rossi, Ishak Yusuf, Pierluigi Mauri, Dario Di Silvestre, Stefano Grolli, Maria Luisa Torre	6
5	Evaluation of a novel mechanical device for the production of micro-fragmented adipose tissue for veterinary regenerative medicine: A proof-of-concept	Priscilla Berni, Valentina Andreoli, Gaia Nina Henriette Di Pasquale, Gabriele Scattini, Luisa Pascucci, Virna Conti, Roberto Ramoni, Martina Pellegrini, Giuseppina Basini, Maurizio Del Bue, Gian Paolo Squassino, Francesca Paino, Paolo Pirazzoli, Antonio Bosetto, Stefano Grolli	7
6	Freeze-dried mesenchymal stem cell secretome loaded on decellularized dermis to improve wound regeneration	Edoardo Bertania, Angelo Modena, Elia Bari, Marta Cecilia Tosca, Lorena Segale, Lorella Giovannelli, Giovanni Sesana, Maria Luisa Torre	8
7	Small extracellular vesicles can reflect tumor invasiveness in patient-derived diffuse midline glioma organoids	Timea Böröczky, Gabriella Dobra, Matyas Bukva, Edina Gyukity-Sebestyén, Maria Harmati, Clementine Barry, Peter Horváth, Marie-Anne Debily, Krisztina Buzás	9
8	Antimicrobial activity of platelet extracellular vesicles and releasate on bovine mastitis pathogens: a preliminary study	Amanda Braga, Costanza Spadini, Valentina Andreoli, Nicolò Mezzasalma, Virna Conti, Elia Bari, Roberto Ramoni, Stefano Grolli, Clotilde Silvia Cabassi	10
9	From lab to clinic: *ex vivo* models for biotherapeutic testing in osteoarthritis	Francesca Cadelano, Stefania Niada, Chiara Giannasi, Elena Della Morte, Nicolò Rossi, Laura Mangiavini, Giuseppe Talò, Anna Teresa Brini	11
10	Isolation and characterization of equine colostrum-derived mesenchymal stromal cells: a potential resource for veterinary regenerative medicine	Angelita Capone, Barbara Merlo, Fabiana Begni, Eleonora Iacono	11
11	Mesenchymal stem cells mediated modulation of the metastatic microenvironment in pancreatic cancer: a step toward clinical translation	Paolo Riccardo Camisa, Benedetta Ferrara, Valeria Lanci, Alessia Solcia, Santi Rapisarda, Antonio Citro, Chiara Gnasso, Valentina Zamarian, Chiara Ceriani, Valentina Coccè, Andrea Annoni, Augusto Pessina, Stefano Crippa, Lorenzo Piemonti	12
12	Good manufacturing practice-derived human liver stem cell extracellular vesicles attenuate *in vivo* liver fibrosis	Elena Ceccotti, Veronica Dimuccio, Massimo Cedrino, Chiara Pasquino, Maria Beatriz Herrera Sanchez, Cristina Grange, Federico Figliolini, Giorgio Nicolò, Selene Limoncelli, Giulio Mengozzi, Giulia Gioiello, Marta Tapparo, Fabio Cattelino, Giovanni Camussi, Valentina Fonsato, Stefania Bruno	13
13	Devitalized microfragmented fat stimulates early adipogenesis and increases UCP1 expression in mesenchymal stromal cells	Valentina Coccè, Sara Missaglia, Eleonora Martegani, Daniela Tavian, Luisa Doneda, Barbara Manfredi, Giulio Alessandri, Aldo Bruno Giannì, Emilio Ciusani, Carlo Tremolada, Francesca Paino, Augusto Pessina	14
14	Use of factors released from human adipose stem cells for ovarian tissue regeneration	Giorgia D'Addato, Nicola Bertani, Margherita Zipponi, Davide Brusa, Alessandra Camboni, Antonella Camaioni, Gina La Sala, Marie-Madeleine Dolmans, Francesca Gioia Klinger	15
15	Mammary adipose tissue-derived mesenchymal stem cells in breast cancer microenvironment: the role of versican proteoglycan	Federica D'Alterio, Alessia Parascandolo, Michele Francesco Di Tolla, Lorenza Zinna, Giusy Ferraro, Serena Cabaro, Pietro Formisano, Vittoria D' Esposito	16
16	Combined biophysical influence on mesenchymal stem cell fate in 3D engineered trabecular long-bone microenvironments	Farah Daou, Ranveer Kaur, Stefano Gabetti, Beatrice Masante, Eleonora Zenobi, Carlotta Achille, Elisa Scatena, Simone Israel, Cristina Bignardi, Diana Massai, Andrea Cochis, Lia Rimondini	17
17	Do polystyrene nanoplastics affect the biological features of dog mesenchymal stromal cells *in vitro*?	Paolo Di Lorenzo, Martina Tambassi, Melissa Berni, Virna Conti, Simona Bussolati, Roberto Ramoni, Erika Scaltriti, Giuseppina Basini, Stefano Grolli	18
18	Treating keratoconjunctivitis sicca with canine mesenchymal stromal cells	Kristina Dojchinovska, Caterina Morera, Alessia Sulla, Gabriele Scattini, Luisa Pascucci, Rolando Arcelli	19
19	Proteomic comparative analysis between equine amniotic mesenchymal stromal cells and their extracellular vesicles	Giulia Gaspari, Alessio Soggiu, Fausto Cremonesi, Anna Lange-Consiglio	19
20	Secretome and extracellular vesicle signatures in bone marrow-derived mesenchymal stromal cells after expansion in standard and next-generation media	Giulio Grieco, Simona Piccolo, Enrico Ragni, Laura de Girolamo	20
21	From mesenchymal stem cells to bone: the role of biomechanical and electromagnetic stimulation in guiding MSC differentiation	Ranveer Kaur, Farah Daou, Stefano Gabetti, Beatrice Masante, Eleonora Zenobi, Carlotta Achille, Elisa Scatena, Simone Israel, Cristina Bignardi, Diana Massai, Andrea Cochis, Lia Rimondini	21
22	The role of extracellular vesicles isolated from mesenchymal stromal cells in myofiber regeneration	Aurora Longhin, Valentina Gatta, Gabriella Teti, Chiara Sassoli, Flaminia Chellini, Alessia Tani, Martina Parigi, Rachele Garella, Francesco Palmieri, Roberta Squecco, Monica Mattioli Belmonte, Caterina Licini, Alessandra La Contana, Sandra Zecchi-Orlandini, Mirella Falconi	22
23	Low-intensity pulsed ultrasound as a promising tool for modulating cartilage regeneration	Cristina Manferdini, Enrico Lenzi, Elena Gabusi, Diego Trucco, Andrea Cafarelli, Lorenzo Vannozzi, Giovanni D'Atri, Leonardo Ricotti, Gina Lisignoli	23
24	Mesenchymal stromal cells-derived extracellular vesicles as carriers for paclitaxel delivery	Angela Marcianti, Eleonora Spampinato, Sara Nava, Giulia Maria Stella, Simona Pogliani, Simona Frigerio, Mariangela Papa, Fabio Moda, Federico Angelo Cazzaniga, Angelo Guido Corsico, Catia Traversari, Daniela Lisini	24
25	Differing responses of adipose-derived and bone marrow-derived mesenchymal stem cells during osteogenic differentiation	Elisa Mazzoni	25
26	Lyosecretome from mesenchymal stem cells for the treatment of bronchopulmonary dysplasia: production and biological activity *in vitro*	Angelo Modena, Elia Bari, Chiara Catozzi, Claudia Moscheni, Simona Turi, Rossana Rossi, Dario Di Silvestre, Francesca Ricci, Gino Villetti, Maria Luisa Torre	25
27	SDH-deficient GIST modeling using genome-edited iPSC-derived mesenchymal stem cells	Ilenia Motta, Livia Gozzellino, Alice Costa, Margherita Nannini, Milena Urbini, Maria Concetta Nigro, Gianandrea Pasquinelli, Maria Abbondanza Pantaleo, Annalisa Astolfi	26
28	Study of the potential effects of different bioglass formulations on the cross-talk between ASCs and HMECs	Clarissa Orrico, Riccardo Pedraza, Alessandro Mosca Balma, Sara Meinardi, Ilaria Roato, Federico Mussano	27
29	The crosstalk between adipose-derived mesenchymal stem cells and microvascular endothelial cells in different culture conditions	Riccardo Pedraza, Clarissa Orrico, Alessandro Mosca Balma, Sara Meinardi, Ilaria Roato, Federico Mussano	28
30	Design and characterisation of a 3D *in vitro* model using human mesenchymal stromal cells for skin wound healing	Federica Re, Luciana Sartore, Elisa Borsani, Chiara Pasini, Federica Trenta, Rosalba Monica Ferraro, Camillo Almici, Andrea Bianchetti, Domenico Russo	29
31	Human plasma creates a physiologic environment to select stem cells from bone marrow stromal cells	Alessia Repetto, Anita Muraglia, Ranieri Cancedda, Gilberto Filaci, Maddalena Mastrogiacomo	30
32	Genome analysis of healthy dental pulp stem cells and periodontal ligament stem cells	Ilaria Roato, Clarissa Orrico, Riccardo Pedraza, Alessandro Mosca Balma, Giacomo Baima, Mario Aimetti, Federico Mussano	30
33	Effect of conditioned medium from human amniotic mesenchymal stromal cells on inflammasome activation in M1 macrophages	Marta Rossi, Marta Magatti, Pietro Romele, Elsa Vertua, Elisabetta Giuzzi, Alice Paini, Anna Pasotti, Silvia De Munari, Antonietta Rosa Silini, Ornella Parolini	31
34	Inward rectifier and calcium-activated potassium currents in amniotic fluid-derived cells	Paola Sabbatini, Sabrina Cipriani, Andrea Biagini, Luana Sallicandro, Flora Ballarino, Cataldo Arcuri5, Rita Romani, Paolo Prontera, Alessandra Mirarchi, Rosaria Gentile, Diletta Del Bianco, Elko Gliozheni, Sandro Gerli, Irene Giardina, Maurizio Arduini, Alessandro Favilli, Antonio Malvasi, Andrea Tinelli, Bernard Fioretti	32
35	Development of a potency assay to evaluate the immunomodulatory potential of canine mesenchymal stromal cells	Gabriele Scattini, Martina Pellegrini, Alessia Sulla, Giulio Severi, Kristina Dojchinovska, Monica Cagiola, Luisa Pascucci	33
36	Engineered extracellular vesicles from mesenchymal stromal cells as nano-shuttles for viral angiogenic oligopeptides	Gabriele Scattini, Giulia Venneri, Cinzia Giagulli, Giulio Alessandri, Luisa Pascucci	34
37	*In vitro* comparison of immunomodulatory properties of mesenchymal stromal cells from human term placenta	Alessandra Spanò, Elsa Vertua, Pietro Romele, Patrizia Bonassi Signoroni, Antonietta Rosa Silini, Ornella Parolini	34
38	2.5D analysis of 3D spheroids for cancer stem cell isolation	Mariachiara Stellato, Vágó Pal, Akos Diosdi, Maria Harmati, Daniel Remondini, Nicola Normanno, Gastone Castellani, Filippo Piccinini, Peter Horvath	35
39	Validation of cryopreserving media for canine mesenchymal stromal cells: a further step toward therapy	Alessia Sulla, Gabriele Scattini, Luisa Pascucci, Ida Duprez, Gianluca Moretti	36
40	Donor sites and harvesting techniques affect miRNA cargos of extracellular vesicles released by human adipose-derived mesenchymal stromal cells	Michela Maria Taiana, Caterina Visconte, Alessandra Colombini, Paola De Luca, Enrico Ragni, Laura de Girolamo	37
41	Feasibility study on the differentiation of mesenchymal stromal cells into fibroblasts for skin regenerative medicine applications	Federica Trenta, Rosalba Monica Ferraro, Elisa Borsani, L. Assoni, Camillo Almici, Andrea Bianchetti, Alessia Cavalleri, Silvia Mutti, Alessandro Leoni, Luca Garufio, Besjana Xhahysa, Simona Bernardi, S. Giliani, Domenico Russo, Federica Re	38
42	Use of the combination of microfat and platelet-rich plasma in a dog with open fracture and tissue loss	Gian Luigi Vannucci, Priscilla Berni, Stefano Grolli, Maurizio Del Bue	39
43	Mesenchymal stromal cell secretome and its potential in the treatment of polycystic ovary syndrome	Alessia Ventura, Maria Assunta Ucci	39
44	Umbilical cord-derived mesenchymal stromal cells and hyaluronic acid-based biomaterials: optimizing cell delivery for enhanced therapeutic persistence	Laura Zocca, Daniela Catanzaro, Martina Bernardi, Elena Merotto, Luisa Galla, Anna Merlo, Francesca Elice, Elisa Zolpi, Cosimo Bleve, Salvatore Fabio Chiarenza, Giuseppe Astori, Martina Piccoli	40

## 1. Impact of platelet-rich plasma on gene expression in bovine endometrial mesenchymal stem cells infected with bovine gammaherpesvirus type 4 and exposed to lipopolysaccharide

Valentina Andreoli^1^, Sofia Lopez^2^, Santiago Delgado^3^, Sandra Perez^4^, Susana Pereyra^2^, Florencia Romeo^2^, Stefano Grolli^5^, Andrea Elizabeth Verna^2^

^1^Department of Agricultural, Forest and Food Sciences, University of Torino, Torino, Italy. ^2^Grupo de Salud Animal, Instituto de Innovación para la Producción Agropecuaria y Desarrollo Sostenible, Balcarce, Argentina. ^3^Facultad de Ciencias Agrarias, Universidad Nacional de Mar del Plata, Mar del Plata, Argentina. ^4^Facultad de Ciencias Veterinarias, Universidad Nacional del Centro de la Provincia de Buenos Aires, Tandil, Argentina. ^5^Department of Veterinary Sciences, University of Parma, Parma, Italy.

Objective: Uterine diseases in cattle are often linked to bacterial infections, with various pathogens identified in the uterine lumen. Bovine gammaherpesvirus type 4 (BoGHV-4) is prevalent in parts of Argentina and has been implicated in postpartum uterine diseases. This study aims to investigate the gene expression profile of bovine endometrial cells (BECs) in response to BoGHV-4 infection in the presence of lipopolysaccharide (LPS) and to evaluate the therapeutic potential of platelet-rich plasma (PRP).

Materials and methods: BECs were isolated from healthy bovine endometrial tissue and cultured in MEM-E supplemented with antibiotics, antimycotics, and fetal bovine serum (FBS). The 07-435 strain of BoGHV-4, isolated from a cow with a history of abortion, was propagated in the MDBK cell line. PRP was prepared from donor cattle blood at a final concentration of 1 × 10^9^ ± 0.2 × 10^9^ platelets/mL. BECs were cultured in media containing either 10% FBS or 10% PRP, infected with BoGHV-4, and subsequently incubated with LPS 24 h post-infection (hpi) to simulate co-infection. The gene expression of TLR4, TNF-α, IL-8, IFN-γ, and BoGHV-4 IE2 was evaluated at 4, 12, 24, and 48 hpi using RT-qPCR on both cells and supernatants (*n* = 3). Statistical analyses were conducted in R using a completely randomized design, comparing the effects of treatments (virus, LPS, and LPS + virus) across time points. Fisher’s test with Bonferroni correction was used for multiple comparisons.

Results: Our findings show that TLR4 and TNF-α expression significantly increased in FBS-cultured BECs upon LPS stimulation, with a stronger effect in BoGHV-4-infected cells. In PRP-cultured cells, changes were less pronounced, with TNF-α expression showing inhibition at 48 h. PRP delayed IL-8 induction and enhanced IFN-γ expression, which may contribute to preserving uterine tissue integrity by mitigating damage associated with co-infections. IE2 expression showed a slight increase at 24 h in PRP cultures, suggesting a potential regulatory effect of PRP on viral replication dynamics.

Conclusions: These findings emphasize PRP’s potential as an effective anti-inflammatory agent for managing uterine diseases, offering a promising alternative to conventional antibiotic treatments. However, further research is needed to elucidate the underlying molecular mechanisms and to optimize its clinical application, ensuring both efficacy and safety in therapeutic settings.

## 2. Case report: treatment of ununited anconeal process (UAP) with microfragmented fat in a dog

Alessandro Guglielmo Aspesi^1^, Maurizio Del Bue^2^, Priscilla Berni^3^, Stefano Grolli^3^

^1^Veterinary Practitioner, Cavaria con Premezzo, Italy. ^2^Veterinary Practitioner, Parma, Italy. ^3^Department of Veterinary Science, University of Parma, Parma, Italy.

Introduction: The Ununited Anconeal Process (UAP) is an alteration that occasionally occurs in rapidly growing purebred dogs. In such cases, the ulna exhibits a fourth ossification center at the level of the anconeal process, rather than the usual three ossification centers (one diaphyseal and two epiphyseal). When present, these centers generally weld together by six months of the dog’s life. However, if this center fails to unite within this time period, it is diagnosed as Ununited Anconeal Process (UAP) and is referred to as Elbow Dysplasia.

Material and methods: An 8-month-old English Mastiff dog presented with left elbow UAP (in association with bilateral Fragmented Coronoid Process and severe bilateral hip dysplasia) at preventive X-ray checks. The dog exhibited lameness of grade II on the left front leg associated with light joint effusion. Palpation revealed intense pain, particularly during maximum elbow extension. Following a thorough evaluation of the available surgical options, the decision was taken to proceed with an intra-articular infiltration of microfragmented fat (microfat). The whole procedure was performed under general anesthesia. Adipose tissue was harvested from the falciform ligament and then immediately fragmented using Tissue-Grinder (Hydra srl, Mirandola). Three milliliters of microfat were administered into the affected joint.

Results: In the first two days following treatment, there was a marked exacerbation of the patient’s lameness. However, this condition exhibited a swift regression over the ensuing days. The patient was placed in a state of rest for a duration of six weeks, after which the dog was permitted to engage in a progressive form of light walking activity. After 75 days, radiographic check revealed that the ununited fragment had undergone complete integration with the ulna.

Conclusions: Non-union of the anconeal process does not resolve spontaneously in 8-month-old dogs and is a causative factor for arthritic processes. In the treated patient, the microfragmented tissue probably favored the endochondral ossification and subsequent union of the fragments. The present case report suggests the necessity for further studies to evaluate the efficacy of microfat in the treatment of UAP in dogs.

## 3. Extracellular vesicles isolated from mesenchymal stromal cells loaded with doxorubicin: the proof of concept for new osteosarcoma treatment

Alessia Santa Giovanna Banche Niclot^1^, Camilla Francesca Proto^1^, Federico Divincenzo^1^, Francesco Barbero^2^, Ivana Fenoglio^2^, Chiara Scarpa^3^, Alessandro Barge^3^, Francesca Paino^4^, Ivana Ferrero^1^, Elisa Tirtei^1^, Katia Mareschi^5^, Franca Fagioli^5^

^1^Stem Cell Transplantation and Cellular Therapy Laboratory, Paediatric Onco-Haematology Division, Regina Margherita Children’s Hospital, City of Health and Science of Turin, Torino, Italy. ^2^Department of Chemistry, University of Turin, Turin, Italy. ^3^Department of Drug Science and Technology, University of Turin, Turin, Italy. ^4^CRC StaMeTec, Department of Biomedical, Surgical and Dental Sciences, University of Milan, Milan, Italy. ^5^Department of Public Health and Paediatrics, The University of Turin, Torino, Italy.

Introduction: Osteosarcoma (OS) is a rare and aggressive bone tumor mainly affecting children and adolescents. Despite intensive chemotherapy, the prognosis remains poor, especially in metastatic or recurrent cases. Extracellular vesicles (EVs) derived from Mesenchymal Stromal Cells (MSCs) are emerging as promising drug delivery systems. This study presents a proof of concept evaluating the cytotoxic potential of MSC-derived EVs loaded with Doxorubicin (DOXO) as a new drug delivery system.

Materials and methods: MSCs were treated with 10 µg/mL DOXO for 24 h to generate a DOXO-enriched secretome (DOXO-SECR). EVs were then isolated using a 100 kDa molecular weight cut-off (MWCO), initially via Amicon Ultra centrifugal filters to validate the method, and subsequently using the KrosFlo KR2i tangential flow filtration (TFF) system under GMP-compliant conditions at the AIFA-accredited Cell Factory. The cytotoxic effects of DOXO-SECR, DOXO-EV-AMICON, and DOXO-EV-TFF were evaluated on three osteosarcoma cell lines (SJSA, MG63, HOS) using an MTT assay after 24 h of exposure, both undiluted and diluted 1:2. Samples were characterized by high-performance liquid chromatography (HPLC) and nanoparticle tracking analysis (NTA).

Results: All three OS cell lines showed sensitivity to free DOXO, with IC50 values at 24 h of 1.31 ± 0.03 µg/mL for SJSA, 1.83 ± 0.05 µg/mL for MG63, and 1.55 ± 0.03 µg/mL for HOS. Treatment with EVs isolated from DOXO-SECR induced cell mortality greater than 50% in all lines. Preliminary HPLC analysis confirmed the presence of DOXO and its metabolites in the EV preparations, with an estimated concentration of approximately 55 ng/mL. These findings suggest a higher cytotoxic effect of EV-associated DOXO compared to the free drug. NTA confirmed the presence of nanoparticles with sizes consistent with EVs: DOXO-SECR (202.9 ± 86.0 nm), DOXO-EV-AMICON (201.7 ± 88.1 nm), and DOXO-EV-TFF (173.4 ± 73.3 nm), indicating comparable size profiles between the two isolation methods.

Conclusions: This proof-of-concept study demonstrates that EVs loaded with DOXO exert significant cytotoxic effects on OS cell lines, surpassing those of free DOXO. Moreover, the reproducibility of EV isolation by both methods - and particularly the successful application of the GMP-compliant TFF system - supports the feasibility of developing DOXO-EV-TFF as a scalable and clinically relevant therapeutic candidate for osteosarcoma treatment within certified cell manufacturing facilities.

## 4. Freeze-dried mesenchymal stem cell secretome for osteoarthritis regenerative medicine: clinical evaluation in dogs and horses

Elia Bari^1^, Priscilla Berni^2^, Maurizio Del Bue^3^, Virna Conti^2^, Valentina Andreoli^2^, Roberto Ramoni^2^, Mario Angelone^2^, Gian Paolo Squassino^3^, Maurizio Rinaldi^1^, Silvia Dotti^4^, Rossana Rossi^5^, Ishak Yusuf^5^, Pierluigi Mauri^5^, Dario Di Silvestre^5^, Stefano Grolli^2^, Maria Luisa Torre^1^

^1^Department of Pharmaceutical Sciences, University of Piemonte Orientale, Novara, Italy. ^2^Department of Veterinary Sciences, University of Parma, Parma, Italy. ^3^Veterinary Practitioner, Parma, Italy. ^4^Istituto Zooprofilattico per la Lombardia e l’Emilia Romagna, Brescia, Italy. ^5^Institute for Biomedical Technologies, Segrate, Italy.

Objective: This study reports veterinary clinical trials testing lyosecretome - a freeze-dried, injectable MSC-secretome obtained through standardized GMP manufacturing - in dogs and horses affected by osteoarthritis (OA).

Materials and methods: MSCs were cultured in a serum-free culture medium for 48 h; supernatants were collected, ultrafiltered (5 kDa) to isolate secretome, and freeze-dried (using mannitol). Lyosecretomes were characterized by proteomics. Five horses presenting naturally occurring fetlock OA were treated with increasing doses of equine lyosecretome (0, up to 2’106 cell equivalents) administered intraarticularly 2 times in total (days 0 and 40). Safety was evaluated through preclinical and postclinical evaluations. Twenty-six dogs suffering from unilateral or bilateral elbow or knee OA were treated with hyaluronic acid (HA, CTR) or lyosecretome (2’106 cell equivalents) intraarticularly injected at days 0 and 40. Safety and efficacy were assessed through orthopaedic evaluations and the filling of a questionnaire containing the validated Helsinki Chronic Pain Index (HCPI). In all cases, lyosecretome was administered using HA as a vehicle.

Results: For both horses and dogs, no systemic adverse reactions were observed in any patients, even after the second administration. In dogs, both treatment and CTR improved OA; however, for dogs with severe OA, there is a significant effect in favor of the lyosecretome treatment when considering lameness evaluation, especially when focusing on the most severe lameness grades (from 3 to 5). Regarding HCPI, both CTR and lyosecretome treatments showed an improvement over time with 3 of the 11 questions, significantly in favor of the lyosecretome treatment: a better improvement was found for the questions “dog’s willingness to walk”, “dog’s willingness to play”, and “dog’s ease of movement after major activity or heavy exercise”. Proteomic analyses supported these efficacy findings, revealing lyosecretome proteins involved in immune response (FTH1, VTN, PDIA3, RTN4, TFRC) and inflammation (Galectin-1), as well as proteins critical for cartilage regeneration and protease inhibition (e.g., lumican, decorin, biglycan, collagen type I proteins) and those with antioxidant activity.

Conclusions: The results support the beneficial effect of MSC-secretome in OA and highlight insights relevant to human patients, as there is a close analogy in OA pathophysiology between human and veterinary populations.

## 5. Evaluation of a novel mechanical device for the production of micro-fragmented adipose tissue for veterinary regenerative medicine: a proof-of-concept

Priscilla Berni^1^, Valentina Andreoli^1^, Gaia Nina Henriette Di Pasquale^1^, Gabriele Scattini^2^, Luisa Pascucci^2^, Virna Conti^1^, Roberto Ramoni^1^, Martina Pellegrini^3^, Giuseppina Basini^1^, Maurizio Del Bue^4^, Gian Paolo Squassino^5^, Francesca Paino^6^, Paolo Pirazzoli^7^, Antonio Bosetto^7^, Stefano Grolli^1^

^1^Department of Veterinary Medical Science, University of Parma, Parma, Italy. ^2^Department of Veterinary Medicine, University of Perugia, Perugia, Italy. ^3^Istituto Zooprofilattico Sperimentale dell’Umbria e delle Marche “Togo Rosati”, Perugia, Italy. ^4^Veterinary Practitioner, Parma, Italy. ^5^Studio Tecnico Veterinario, Asti, Italy. ^6^CRCStaMeTec, Department of Biomedical, Surgical and Dental Sciences, University of Milan, Milan, Italy. ^7^Hydra srl, Mirandola, Italy.

Objective: Therapies based on mesenchymal stromal cells (MSCs) have become one of the most significant advancements in veterinary regenerative medicine. The isolation of MSCs is usually performed by enzymatic digestion and requires variable times for cell expansion. In addition, these procedures need to be performed in specialized laboratory facilities. Among the different therapeutic approaches based on MSCs, the use of SVF and micro-fragmented fat (microfat) represents an attractive alternative for the veterinary surgeon. They are rich sources of cells and growth factors and offer several practical advantages as ready-to-use products, avoiding the time-consuming delays that characterize the *in vitro* expansion of autologous cells. Furthermore, recent clinical studies support their safety and efficacy in the treatment of musculoskeletal disorders and wound healing.

Materials and methods: In this scenario, the present work aimed to characterize the canine micro-fragmented adipose tissue obtained by a new mechanical device (T-Grinder™), sterile and ready-to-use for clinical practice. The biological properties of the micro-fragmented fat were evaluated by analyzing cell viability and growth curves. Phenotypic characterization was performed by flow cytometry and trilineage differentiation, while the expression of a panel of genes involved in MSC therapeutic features was analyzed by real-time PCR. Furthermore, the conditioned medium derived from microfat culture was evaluated for its ability to promote autologous and allogeneic MSC vitality.

Results: No differences were observed between MSCs obtained through mechanical fragmentation and those derived from collagenase digestion of adipose tissue. Furthermore, conditioned medium derived from microfat culture was able to support canine MSC growth, replacing the supplementation with fetal bovine serum.

Conclusions: The device described in the present study enables the preparation of ready-to-use microfat from perivisceral adipose tissue. Microfat-derived MSCs demonstrate equivalent phenotypic characteristics and replicative and differentiation capacities to those obtained by enzymatic digestion. Moreover, conditioned medium prepared from microfragmented fat supports cell viability when used as a substitute for fetal bovine serum, suggesting its possible use for the preparation of xenogeneic-free MSC cultures suitable for clinical application.

## 6. Freeze-dried mesenchymal stem cell secretome loaded on decellularized dermis to improve wound regeneration

Edoardo Bertania^1^, Angelo Modena^1^, Elia Bari^1^, Marta Cecilia Tosca^2^, Lorena Segale^1^, Lorella Giovannelli^1^, Giovanni Sesana^2^, Maria Luisa Torre^1^

^1^Department of Pharmaceutical Sciences, University of Piemonte Orientale, Novara, Italy. ^2^Grande Ospedale Metropolitano Niguarda, Milano, Italy.

Objective: This study couples decellularized dermis with lyosecretome - freeze-dried, injectable MSC-secretome obtained through standardized GMP manufacturing - to enhance wound regeneration potential.

Materials and methods: Dermis samples (collected from 4 different donors, 18-70 years) were decellularized using CHAPS and SDS as surfactants and then freeze-dried. Each scaffold was characterized in terms of morphology (SEM), histology (H&E), physical-chemical properties (FTIR and TGA), swelling, and *in vitro* cytocompatibility (by MTT). Non-decellularized freeze-dried dermis (NT) was used as a control. MSCs were cultured in a serum-free culture medium for 48 h; the supernatants were collected, ultrafiltered (5 kDa) to isolate the secretome, and freeze-dried (using mannitol). Lyosecretome was characterized by particle size, protein content, and lipid content before being loaded on the scaffolds by adsorption. The release of secretome proteins and lipids from the scaffold was assessed for up to 48 h.

Results: All protocols were effective in decellularizing the dermis without altering the microstructure of the ECM. As confirmation of the effective decellularization, numerical data from FTIR spectra showed a marked lowering of lipidic content. TGA revealed no variations in thermally activated degradation of collagen fibres between decellularized and NT. All dermis scaffolds were able to absorb water in abundance, with swelling up to 400%. Fibroblasts, when seeded on dermis scaffolds, were able to proliferate; H&E staining and SEM confirmed proper scaffold colonization, while confocal microscopy showed that the cells successfully adapted to the tissue architecture, adopting an elongated shape along with the direction of the ECM fibers. The release of lyosecretome proteins was sustained up to 20 h and higher from the NT sample than the decellularized ones (likely due to reduced protein adsorption resulting from the presence of cells in NT). For the lipids, the release was completed almost immediately (< 6 h), likely due to the lower tendency of EVs, being lipidic, to adsorb onto dermal components. Elaboration of the release’s kinetic models revealed that the release of proteins from dermis scaffolds is primarily governed by diffusion.

Conclusions: This work provides an *in vitro* proof-of-concept of using a decellularized dermis scaffold to deliver MSC-secretome in wounds, paving the way for further *in vivo* efficacy tests.

## 7. Small extracellular vesicles can reflect tumor invasiveness in patient-derived diffuse midline glioma organoids

Timea Böröczky^1,2,3^, Gabriella Dobra^1^, Matyas Bukva^1^, Edina Gyukity-Sebestyén^1^, Maria Harmati^1^, Clementine Barry^4^, Peter Horváth^1,5,6^, Marie-Anne Debily^3^, Krisztina Buzás^1,2^

^1^Institute of Biochemistry, HUN-REN Biological Research Centre, Szeged, Hungary. ^2^Department of Immunology, University of Szeged, Szeged, Hungary. ^3^Doctoral School of Interdisciplinary Medicines, University of Szeged, Szeged, Hungary. ^4^Vectorologie et Nouvelles Thérapies Anticancéreuses, Gustave Roussy, Université Paris-Saclay, Villejuif, France. ^5^Institute of AI for Health, Helmholtz Zentrum München, Neuherberg, Germany. ^6^Institute for Molecular Medicine Finland (FIMM), University of Helsinki, Helsinki, Finland.

Objective: Diffuse midline gliomas (DMGs) are among the most aggressive pediatric brain tumors, characterized by extensive invasion and a poor survival rate (< 12 months). Due to their involvement in critical brain structures and infiltrative nature, biopsies carry high risks, and surgical resection is severely limited. The heterogeneity in invasive potential among patients impacts disease progression and therapeutic response. In this study, we aim to develop a novel extracellular vesicle-based approach as a less-invasive method to assess tumor invasiveness using patient-derived DMG organoids, potentially aiding therapeutic decision making.

Materials and methods: Our collaboration partners designed and patented (UE EP20306031.4) a 3D tumoroid assay for short-term culturing of DMG stem cells in a hydrogel-based extracellular matrix to predict metastatic behavior. Supernatants were obtained from 3D tumoroids generated from tumor cells of five low- and four highly invasive DMG patients. Small extracellular vesicles (sEVs) were isolated via size-exclusion chromatography (IZON; qEV1 35 nm) and quantified using nanoparticle tracking analysis (Malvern; NanoSight300). Raman spectroscopy (Bruker; Senterra II) with a calcium fluoride substrate was used to assess the overall composition of the isolated sEVs. Data were analyzed using Orange Data Mining software (version 3.36).

Results: Tumoroids derived from low-invasive DMG samples secreted significantly higher amounts of sEVs compared to those from highly invasive samples (*P* = 0.0109). Moreover, Raman spectral analysis of sEVs revealed distinct molecular fingerprints that correlate with tumor invasiveness, allowing clear differentiation between high- and low-invasive gliomas. These findings suggest that the molecular composition of tumor-derived vesicles varies with the invasion capacity of the donor cells and can be effectively detected using Raman spectroscopy.

Conclusions: This study demonstrates that sEVs derived from patient-specific glioma organoids carry distinct molecular signatures that can reflect tumor invasiveness. This tumoroid-based approach could serve as a foundation for extending the analysis to clinical samples, such as cerebrospinal fluid-derived vesicles from glioma patients. Moreover, this work is relevant not only to oncology, but also to regenerative medicine applications involving organoids with mesenchymal stem cells.

Fundings: EUGLOHRIA101017572, TKP2021-EGA-09, OTKA- K143255, EKÖP-24-3SZTE-318.

## 8. Antimicrobial activity of platelet extracellular vesicles and releasate on bovine mastitis pathogens: a preliminary study

Amanda Braga^1^, Costanza Spadini^1^, Valentina Andreoli^1^, Nicolò Mezzasalma^1^, Virna Conti^1^, Elia Bari^2^, Roberto Ramoni^1^, Stefano Grolli^1^, Clotilde Silvia Cabassi^1^

^1^Department of Veterinary Science, University of Parma, Parma, Italy. ^2^Department of Pharmaceutical Sciences, University of Piemonte Orientale, Novara, Italy.

Objective: The rise of antimicrobial resistance in human and veterinary medicine calls for urgent solutions. Recent research has explored the antimicrobial properties of Mesenchymal Stromal Cells (MSCs) and Platelet-Rich Plasma (PRP). The present *in vitro* study focused on a cell-free approach, evaluating the antibacterial effects of bovine activated-platelet derived releasate (PR) and isolated platelet extracellular vesicles (iPEV) against bovine mastitis bacteria: Staphylococcus aureus, methicillin-sensitive and methicillin-resistant (MRSA) strains, Streptococcus agalactiae, and Escherichia coli.

Materials and methods: To prepare PR, bovine pure-PRP was centrifuged to discard the plasma, and platelets were resuspended in phosphate-buffered saline (PBS) at a concentration of 6 × 10^9^ platelets/mL. Platelets were activated by adding calcium gluconate and calcimycin. The following day, the supernatant was centrifuged to remove cell debris. To prepare iPEV, an aliquot of PR was ultracentrifuged at 100,000 × *g* for 1 h. The extracellular vesicles were resuspended in PBS to achieve a final iPEV concentration equivalent to 15 × 10^9^ platelets/mL. Ipev particle size distribution and concentration were evaluated using NTA. The antimicrobial activity of the compounds (*n* = 4) was evaluated through Minimum Inhibitory Concentration (MIC) assay on ATCC bacterial strains.

Results: Preliminary results showed that both the tested compounds exerted an inhibitory effect toward Gram-positive bacteria (both antimicrobial-sensitive and resistant strains). Mean MIC of the compounds on S. aureus: PR = 27.4%, iPEV = 20.8%; on MRSA: PR = 25%, iPEV = 15.9%; on S. agalactiae: PR = 4.63%, iPEV = 6.19%. However, they were not effective against E. coli. In most cases, low-temperature storage (refrigeration and freezing) seemed to decrease antimicrobial activity. No significant difference was detected between the MIC of fresh PR and iPEV.

Conclusions: This work provides new insights into the potential use of platelet-derived releasate and EVs as alternatives to antibiotics. Considering these preliminary findings, platelet derivatives appear to be effective against some of the major Gram-positive mastitis pathogens. Future research should determine whether iPEV and PDS exert their antibacterial activity *in vivo* as well and should investigate the combination of iPEV and antibiotics. Finally, evaluation of alternative storage methods, such as lyophilization, should be explored to provide off-the-shelf products.

## 9. From lab to clinic: *ex vivo* models for biotherapeutic testing in osteoarthritis

Francesca Cadelano^1,2^, Stefania Niada^2^, Chiara Giannasi^1,2^, Elena Della Morte^2^, Nicolò Rossi^3^, Laura Mangiavini^3,4^, Giuseppe Talò^5^, Anna Teresa Brini^1^

^1^Department of Biomedical, Surgical and Dental Sciences, University of Milan, Milan, Italy. ^2^Laboratory of Biotechnological Applications, IRCCS Ospedale Galeazzi-Sant’Ambrogio, Milan, Italy. ^3^Équipe Universitaria di Ortopedia Rigenerativa e Ricostruttiva (EUORR), IRCCS Ospedale Galeazzi- Sant’Ambrogio, Milan, Italy. ^4^Department of Biomedical Sciences for Health, University of Milan, Milan, Italy. ^5^Cell and Tissue Engineering Laboratory, IRCCS Ospedale Galeazzi-Sant’Ambrogio, Milan, Italy.

Objective: The primary aim of this study was to establish reliable *ex vivo* models to evaluate the efficacy of biotherapeutics in pathological settings. Specifically, we investigated the potential of adipose-derived stem/stromal cell (ASC)-derived secretome (or conditioned medium, CM) to mitigate inflammation and degenerative processes associated with osteoarthritis (OA).

Materials and methods: *Ex vivo* experiments were conducted using human cartilage, osteochondral interface, and synovial membrane explants. An inflammatory phenotype was induced with OA-relevant cytokines, namely TNFα and/or IL-1β. Control and stimulated groups were compared with those treated with CM or primed CM (pCM), a “potentiated” CM derived from cytokine-primed ASCs. Markers relevant to tissue viability, catabolic processes, and inflammation were assessed. For cartilage, MMP activity, GAG release, and specific gene and protein expression were analyzed. In the synovial membrane, nitric oxide production, COX2, and PGE2 expression served as key inflammatory markers.

Results: The results demonstrated that all *ex vivo* explants remained viable for several days and responded to inflammatory cytokines. Cartilage explants provided rapid and informative insights for identifying the most effective cytokine stimulation and demonstrated the effect of CM on catabolic MMP activity. The osteochondral interface model proved particularly effective, enabling the separation of cartilage and bone compartments for localized treatment and analysis. CM, both standard and primed, proved effective in modulating catabolic activity in the osteochondral model as well. The synovial membrane model revealed CM action in reducing several inflammatory markers.

Conclusions: In conclusion, this study highlights the utility of *ex vivo* models as robust platforms for replicating OA features and testing biotherapeutics. CM demonstrated the ability to reduce catabolic activity and inflammation, supporting its therapeutic potential in OA treatment. These findings suggest that *ex vivo* models can be effectively integrated into drug testing pipelines, and further studies are warranted to validate these results and facilitate the clinical translation of CM.

## 10. Isolation and characterization of equine colostrum-derived mesenchymal stromal cells: a potential resource for veterinary regenerative medicine

Angelita Capone, Barbara Merlo, Fabiana Begni, Eleonora Iacono

Dipartimento di Scienze Mediche Veterinarie (DIMEVET), Università di Bologna, Ozzano dell’Emilia, Italy.

Objective: Colostrum, the first secretion of the mammary gland, is a nutrient-rich fluid crucial for neonatal survival. In equine species, it serves as the sole source of immunoglobulins due to the epitheliochorial placenta. Beyond its immunological role, colostrum has emerged as a promising, non-invasive source of bioactive factors, including Mesenchymal Stromal Cells (MSCs). This study represents the first attempt to isolate and characterize MSCs from equine colostrum (C-MSCs) and assess their potential for veterinary regenerative medicine.

Materials and methods: Colostrum (*n* = 6) was collected immediately postpartum from mares hospitalized for delivery. Samples were centrifuged and recovered cells cultured under standard conditions. Cell growth and clonogenic potential were assessed by population doubling time (PDT) analysis and colony-forming unit (CFU) assay, respectively. MSC identity was confirmed via adhesion (spheroid formation) and migration assays, trilineage differentiation (osteogenic, chondrogenic, adipogenic), and RT-PCR for mesenchymal (CD90, CD73), hematopoietic (CD45, CD34), and immune (MHC-I, MHC-II) markers. Statistical analyses were performed using one-way and two-way ANOVA (*P* < 0.05) to assess individual variability.

Results: C-MSCs displayed plastic adherence and heterogeneous morphology, including spindle-shaped and epithelial-like cells. PDT values varied among samples and 4/6 showed rapid proliferation (< 2 days). CFU assays confirmed clonogenic potential, though significant inter-sample variability was observed (*P* < 0.05). Spheroid formation assays revealed differences in cell-cell adhesion: 4/6 samples formed stable spheroids within four days. Migration assay showed significant variability (*P* < 0.05): 1/6 sample achieved complete wound closure within 72 h, whereas 5/6 samples progressed more slowly, reaching ~30% closure at 96 h. Differentiation assays confirmed trilineage potential and all samples showed positive staining for adipogenic, chondrogenic, and osteogenic differentiation. RT-PCR analysis confirmed MSC marker expression, while hematopoietic markers were absent. MHC-I expression was weak in 5/6 samples, whereas MHC-II was consistently negative.

Conclusions: Data recorded in the present study support equine colostrum as a viable, non-invasive MSC source. However, the observed variability requires validation with a greater number of samples. Moreover, further research is needed to explore C-MSCs’ immunomodulatory properties and their therapeutic application potential.

## 11. Mesenchymal stem cells mediated modulationof the metastatic microenvironment in pancreatic cancer: a step toward clinical translation

Paolo Riccardo Camisa^1^, Benedetta Ferrara^1^, Valeria Lanci^1^, Alessia Solcia^1^, Santi Rapisarda^1^, Antonio Citro^1^, Chiara Gnasso^2^, Valentina Zamarian^1^, Chiara Ceriani^1^, Valentina Coccè^3^, Andrea Annoni^4^, Augusto Pessina^3^, Stefano Crippa^5^, Lorenzo Piemonti^1^

^1^Diabetes Research Institute, Vita-Salute San Raffaele University, Milan, Italy. ^2^Experimental Imaging Centre, IRCCS San Raffaele, Milano, Italy. ^3^Department of Biomedical, Surgical and Dental Sciences, University of Milan, Milan, Italy. ^4^Immune Core, San Raffaele Telethon Institute for Gene Therapy, IRCCS San Raffaele, Milano, Italy. ^5^Division of Pancreatic Surgery, Pancreas Translational & Clinical Research Center, IRCCS San Raffaele Scientific Institute, Milan, Italy.

Objective: Metastatic pancreatic ductal adenocarcinoma (mPDAC) is associated with poor prognosis and chemoresistance, partially because of the complex tumor microenvironment (TME). In this context, Mesenchymal stem cells (MSCs) are a promising tool, given their ability to uptake drugs and target tumors. The aim of this project is to investigate the effects of MSC therapy for the delivery of nab-paclitaxel (n-PTX) in preclinical models of mPDAC.

Materials and methods: Liver metastases were induced by injecting K8484 cells into the portal vein of C57BL/6N mice. Metastatic mice were intraportally injected with syngeneic or allogeneic MSCs. Biodistribution was studied by IVIS using luciferase+ MSCs and RT-PCR. For efficacy, MSCs were loaded with n-PTX and metastatic growth was monitored by MRI and toxicity assessed through biochemical analysis. The immune infiltrate was assessed with IHC and Flow Cytometry. The same experimental setup was used to induce and treat human metastases using BxPC-3 cells in NSG mice.

Results: RT-PCR and IVIS revealed a liver accumulation of MSCs. N-PTX-loaded MSCs reported a significant metastatic growth reduction compared to mice treated with non-loaded MSCs. A lower hematological toxicity was reported using n-PTX-loaded MSCs, compared to free n-PTX. The intraportal injection transiently increased serum transaminases, normalizing within a week. The infusion of MSCs, both syngeneic and allogenic, induced a significant increase in the CD3+ cells within the TME compared to controls, and bulk RNAseq analysis 24 h post MSC+N-PTX injection demonstrated an enrichment in several pathways linked to antitumor immune response. Notably, treatment with syngeneic MSCs reduced macrophage infiltration in the TME compared to controls, whereas allogeneic MSC infusion led to an increase in their presence. The injection of BxPC-3 cells led to the establishment of a xenograft model mimicking human metastasis; in this model, human MSCs loaded with PTX demonstrated a significant reduction in the metastatic progression.

Conclusions: A novel approach employing MSCs as carriers for n-PTX in the treatment of mPDAC was developed. This study evaluated the efficacy, distribution, and toxicity of this cell-based therapy in preclinical models. Notably, the observed changes in the TME suggest potential for combinational therapy with other immune-therapies. Overall, these findings offer valuable insights that may facilitate translation into human clinical trials.

## 12. Good manufacturing practice-derived human liver stem cell extracellular vesicles attenuate *in vivo* liver fibrosis

Elena Ceccotti^1^, Veronica Dimuccio^2^, Massimo Cedrino^3^, Chiara Pasquino^2^, Maria Beatriz Herrera Sanchez^4^, Cristina Grange^1^, Federico Figliolini^2^, Giorgio Nicolò^2^, Selene Limoncelli^5^, Giulio Mengozzi^1,5^, Giulia Gioiello^5^, Marta Tapparo^1^, Fabio Cattelino^3^, Giovanni Camussi^1^, Valentina Fonsato^2,4^, Stefania Bruno^1^

^1^Dipartimento di Scienze Mediche, Università di Torino, Torino, Italy. ^2^CLF, Cell Factory, Università di Torino, Torino, Italy. ^3^Molecular Biotechnology Center Guido Tarone, Torino, Italy. ^4^2i3t, Incubatore per le Imprese, Università di Torino, Torino, Italy. ^5^Laboratorio Baldi e Riberi, Città della Salute e della Scienza, Torino, Italy.

Objective: Human liver stem cells (HLSCs) are a mesenchymal stromal cell (MSC)-like population isolated from adult liver biopsies. HLSCs share key characteristics with MSCs, including phenotype, gene expression profile, and differentiation capabilities. In addition, HLSCs show a specific commitment to hepatic phenotype. Previous studies have demonstrated that HLSCs improve recovery in various experimental models of acute and chronic tissue injuries, and HLSC-derived extracellular vesicles (HLSC-EVs) mimic the therapeutic effects of the cells of origin. The study aims to evaluate whether HLSC-EVs, obtained under good manufacturing practice (GMP) conditions, can influence the progression of liver fibrosis *in vivo*.

Materials and methods: The EV production process was carried out under GMP conditions to generate batches of HLSC-EVs. These batches consisted of multiple cryobags, each containing a concentration of EVs of ≥ 4 × 10^9^ particles/mL, resuspended in 50 mL of freezing solution.

The production process involved two sequential steps:

1. HLSC Cell Stock expansion: starting from a certified Master Cell Bank, HLSCs were expanded to produce and collect the Conditioned Medium.

2. HLSC-EV isolation and formulation: EVs were isolated using Tangential Flow Filtration, followed by filtration and final drug product formulation.

HLSC-EVs were characterized for their immunophenotype, size, integrity, sterility, and biological activity. To assess their therapeutic potential, an *in vivo* model of hepatic fibrosis was developed through bi-weekly intraperitoneal administration of thioacetamide (TAA, 200 mg/Kg) for 8 weeks. EV treatment was initiated after 4 weeks of TAA administration and was performed intravenously once a week for 4 weeks (5 × 10^9^ EVs/ injection). TAA mice were sacrificed at week 8 and biochemical, histological, and molecular analyses were performed.

Results: In TAA mice, EV-treatment attenuated fibrosis development at the histological level. Molecular analyses showed a significant reduction in gene expression levels of key pro-fibrotic markers, such as Collagen IaI, alpha-smooth muscle actin, and transforming growth factor beta. Functionally, EV administration significantly improved plasma levels of alanine aminotransferase and albumin, indicating enhanced liver function.

Conclusions: HLSC-EVs, produced under GMP conditions, display anti-fibrotic effects in a chronic liver disease model, improving liver function and histology.

## 13. Devitalized microfragmented fat stimulates early adipogenesis and increases UCP1 expression in mesenchymal stromal cells

Valentina Coccè^1^, Sara Missaglia^2^, Eleonora Martegani^2^, Daniela Tavian^2^, Luisa Doneda^1^, Barbara Manfredi^1^, Giulio Alessandri^1^, Aldo Bruno Giannì^1^, Emilio Ciusani^3^, Carlo Tremolada^4^, Francesca Paino^1^, Augusto Pessina^1^

^1^CRC StaMeTec, Department of Biomedical, Surgical and Dental Sciences, Università degli Studi di Milano, Milano, Italy. ^2^Laboratory of Cellular Biochemistry and Molecular Biology, CRIBENS, Università Cattolica del Sacro Cuore, Milano, Italy. ^3^Fondazione IRCCS Istituto Neurologico “C.Besta”, Milano, Italy. ^4^Istituto Image, Milano, Italy.

Objective: Adipose tissue is primarily composed of adipocytes, along with mesenchymal stromal/stem cells (MSCs), macrophages, endothelial cells, and extracellular matrix elements. Its rich vascular network and the presence of various molecules, such as cytokines and growth factors, contribute to its function as an endocrine organ, though this requires further investigation. This study aims to evaluate whether microfragmented adipose tissue (MFAT) plays a role in the differentiation of MSCs into adipocytes *in vitro*.

Materials and methods: Human lipoaspirates were obtained from healthy donors who provided informed consent in accordance with the Declaration of Helsinki and were approved by the Institutional Ethical Committee of Milan University (no. 59/15, C.E.UNIMI, 09.11.15). Fat specimens were mechanically microfragmented (MFAT) using the commercial device “Lipogems®” and subjected to freezing-thawing cycles for devitalization. The thawed MFAT was then used to stimulate mesenchymal stem cell (MSC) cultures derived from adipose tissue (AT-MSCs). These results were compared to those obtained using traditional *in vitro* stimulation with an adipogenic medium.

Results: MSCs exposed to MFAT showed early lipid droplet formation after just three days, along with an increase in adipokine expression. A significant overexpression of PPAR gamma coactivator 1 alpha (PPARGC1A) and uncoupling protein 1 (UCP1) was observed in MFAT-stimulated AT-MSCs, suggesting a differentiation pattern consistent with beige/ brown fat. Although these markers were expressed by AT-MSCs at baseline, MFAT stimulation led to a marked increase in their expression.

Conclusions: The regulatory mechanisms of UCP1 transcription are complex and not fully understood, but in brown adipose tissue, the activation of mitogen-activated protein kinase (MAPK) can stimulate lipases, promoting triglyceride hydrolysis and producing fatty acids that activate UCP1. Given that MFAT contains both triglycerides and fatty acids, our model suggests that the triglycerides in MFAT are readily hydrolyzed in the culture medium, increasing fatty acid concentration and activating UCP1. In conclusion, our findings suggest that MSCs have basal UCP1 expression, and that MFAT stimulates its mRNA expression and translation, likely due to the high triglyceride and fatty acid content in MFAT.

## 14. Use of factors released from human adipose stem cells for ovarian tissue regeneration

Giorgia D’Addato^1^, Nicola Bertani^1^, Margherita Zipponi^2^, Davide Brusa^3^, Alessandra Camboni^4^, Antonella Camaioni^5^, Gina La Sala^6^, Marie-.Madaleine Dolmans^2^, Francesca Gioia Klinger^1^

^1^Saint Camillus International University of Health Sciences, Rome, Italy. ^2^Pôle de Gynecologie, Institut de Recherc Experimentale et Clinique, Universite Catholique de Louvain, Brussels, Belgium. ^3^Université catholique de Louvain (UCLouvain), Institute of Experimental and Clinical Research (IREC), Brussels, Belgium. ^4^Department of Pathology, Cliniques Universitaires Saint-Luc, Brussels, Belgium. ^5^Department of Biomedicine and Prevention, Section of Histology and Embryology, University of Rome Tor Vergata, Rome, Italy. ^6^Institute of Biochemistry and Cell Biology, Italian National Research Council (CNR), Monterotondo Scalo, Italy.

Objective: Young women undergoing chemotherapy often experience premature ovarian insufficiency (POI), a medical condition with no treatment available yet. In 2021, our lab successfully reported the first evidence of the efficacy of human adipose-derived stem cells (ASCs) transplantation in prolonging reproductive lifespan in a POI *in vivo* mouse model (1). The aim of this study is to explore the protective ability of the whole ASC secretome on the ovarian reserve and to investigate the content of sEVs. It is important to highlight that different ASC cell lines were used to investigate the potential inter-donor variations.

Materials and methods: ASCs were isolated from two donors (♂-♀), cultured, and their conditioned media (CM) were collected after 1 and 3 days of culture. A POI model was established by culturing ovarian fragments from 4dpp transgenic GFP/c-kit mice *in vitro* in the presence of 10μM phosphoramide mustard (PM) (2). CM obtained from each donor and at different time points were added to the ovarian cultures, and primordial follicle (PMF) survival was assessed by morphological analysis and NOXA mRNA expression over 48 h. The various CM samples were analyzed for their sEV content using nanoparticle tracking analysis (NTA), cytofluorimetry, and transmission electron microscopy (TEM).

Results: After 48 h of culture, PMF survival was higher when ovarian fragments were cultured in the presence of donor♂ CM than when treated with PM alone. No differences were observed between PM and PM+CM♀ groups (% survival: PM 2.43%; ctrl 50.50%, *P* < 0.0001; PM+CM♂1d 27.64%, *P* = 0.0499; PM+CM♂3d 34.76%, *P* = 0.0314; PM+CM♀1d 3.14%; PM+CM♀3d 1.25%). Levels of NOXA mRNA expression confirmed a protective action of the donor♂ CM (2^-ΔCt: PM 0.0117; ctrl 0,0026, *P* = 0.0005; PM+CM♂1d 0.0024, *P* = 0.0006; PM+CM♂3d 0.0052, *P* = 0.0058; PM+CM♀1d 0.0135). Regarding sEVs-CM characterization, NTA showed that both donor ♂ and ♀ CM contain sEVs resembling exosomes according to the size (CM♂1d 131 nm; CM♂3d 147.2 nm; CM♀1d 138.7 nm; CM♀3d 136 nm). Cytofluorimetry analysis also demonstrated that most isolated particles were positive for CD81 and CD9 (CM♂1d 98.3%; CM♂3d 99.6%; CM♀1d 91.8%; CM♀3d 93.7%). Additionally, TEM imaging revealed round, double-membrane structures.

Conclusions: Our findings suggest that although CM counteracts PMF-induced PM depletion, a careful evaluation of CM is necessary, as they are not all equivalent. Then, a comprehensive analysis of their composition is an indispensable basis for the development of treatments for POI patients.

## 15. Mammary adipose tissue-derived mesenchymal stem cells in breast cancer microenvironment: the role of versican proteoglycan

Federica D'Alterio^1^, Alessia Parascandolo^1^, Michele Francesco Di Tolla^1^, Lorenza Zinna^1^, Giusy Ferraro^1^, Serena Cabaro^1^, Pietro Formisano^1^, Vittoria D' Esposito^2^

^1^Department of Translational Medicine, University of Naples, “Federico II”, Naples, Italy. ^2^Institute of Endotypes in Oncology, Metabolism and Immunology “G. Salvatore” (IEOMI)-National Council of Research (CNR), Naples, Italy.

Objective: Mesenchymal stem cells (MSCs) have great potential in regenerative medicine, but their role in supporting disease progression is understudied. Bone marrow-derived MSCs are known to aid breast cancer (BC) progression, yet the local contribution of mammary adipose tissue-derived MSCs (MAT-MSCs) needs to be further investigated. Recently, we have demonstrated that Versican proteoglycan (Vcan) is highly expressed in BC-adipose tissue (BC-AT) compared to healthy AT, correlating with tumoral KI67. This study aims to determine whether MAT-MSCs produce Vcan, how metabolic factors influence its production, and its role in the MAT-MSC and BC cross-talk.

Materials and methods: MAT-MSCs were isolated from MAT biopsies (*N* = 18) from healthy women; Vcan expression levels were analyzed by RT-PCR and ELISA assay. For co-culture experiments, three MAT-MSC isolates with high Vcan expression and three isolates with low Vcan expression were selected and co-cultured with MCF7 BC cell lines. 2D co-cultures were established in transwell systems, after which total RNA was collected and analyzed by RT-PCR. 3D co-cultures were set up in ultra-low attachment wells, and spheroid size was measured using Fiji/ImageJ software on days 3, 6, and 10.

Results: mRNA content and ELISA analysis identified MAT-MSCs as a source of Vcan, with a positive and significant correlation between Vcan expression and patient glycemia (*P* = 0.005). 2D co-culture experiments showed that higher levels of Vcan produced by MAT-MSCs promoted cross-talk with MCF7, leading to increased expression of pro-angiogenic factors (VEGF, *P* < 0.05), inflammatory and fibrotic markers (IL6, αSMA, and FAP, all *P* < 0.05) in MAT-MSCs, as well as elevated levels of matrix metalloproteinase MMP9 (*P* < 0.005) in MCF7. In 3D co-culture experiments, MAT-MSCs with high Vcan expression formed tumor spheroids with larger surface areas compared to MAT-MSCs with low proteoglycan levels (3, 6, and 10 days, all *P* < 0.0001).

Conclusions: MAT-MSCs exert a local action in BC, establishing a cross-talk with BC cells that contributes to a tumor-supporting microenvironment. Vcan secreted by MAT- MSCs has a key role in this interplay and its increase is reminiscent of patient metabolic status.

## 16. Combined biophysical influence on mesenchymal stem cell fate in 3D engineered trabecular long-bone microenvironments

Farah Daou^1^, Ranveer Kaur^1^, Stefano Gabetti^2,3^, Beatrice Masante^2,3^, Eleonora Zenobi^4^, Carlotta Achille^5^, Elisa Scatena^4^, Simone Israel^2,3^, Cristina Bignardi^2,3^, Diana Massai^2,3^, Andrea Cochis^1^, Lia Rimondini^1^

^1^Department of Health Sciences, Center for Translational Research on Autoimmune and Allergic Diseases (CAAD), University of Eastern Piedmont (UPO), Novara, Italy. ^2^Department of Mechanical and Aerospace Engineering and PolitoBIOMed Lab, Politecnico di Torino, Turin, Italy. ^3^Interuniversity Center for the Promotion of the 3Rs Principles in Teaching and Research, Turin, Italy. ^4^Hypatia Research Consortium, Rome, Italy. ^5^E. Amaldi Foundation, Rome, Italy.

Objective: In bone healing, mesenchymal stem cells (MSCs) are recruited, and their osteogenic differentiation is influenced by biochemical and biophysical factors. Extrinsic pulsed electromagnetic field (PEMF) stimulation has been applied to stimulate bone healing and regeneration. As such, the objective of this study was to investigate the influence of various biophysical stimuli on MSC fate within 3D-engineered trabecular long-bone microenvironments.

Materials and methods: Scaffolds. 3D-printed polylactic acid (pla) scaffolds (referred to as p3s3) were designed to mimic the human trabecular bone architecture in physiological conditions and to resemble the morphological features of the ulna, tibia, and femur. Cells. bone marrow-derived mscs (bmscs) were seeded onto the p3s3 scaffolds. Bioreactor. a bioreactor platform that allows for the application of various mechanical stimuli, including fluid flow- induced shear stress induced by direct perfusion with flow rates (q) between 0.006-24 mL/min, and cyclic hydrostatic pressure induced by the intermittent closing of a solenoid- actuated pinch valve with periods (t) between 0.25 to 30 s and duty cycles (dc) between 0% to 100%. moreover, the bioreactor is combinable with a commercial pemf generator (1.5 mt, 75 hz). Culture conditions. trabecular bone tissue-like constructs were cultured for 14 days under either direct perfusion [Q = 0.3 mL/min, referred to as dynamic culture (D)] or combined perfusion and hydrostatic pressure of 15 kPa for 2 h daily, generated by pinch valves operating at T = 10 s and DC = 70% [referred to as rehabilitation program 1 (RP1)].

Results: The introduction of hydrostatic pressure resulted in enhanced cell viability and increased osteogenic differentiation. Furthermore, hydrostatic pressure significantly upregulated genes related to mechanotransduction, indicating its importance in bone tissue engineering. Ongoing studies are exploring the synergistic effects of RP1 and PEMF stimulation (4 h/day), with gene expression analysis to elucidate the role of PEMF in mechanotransduction and osteogenesis.

Conclusions: This study demonstrates the enhanced capabilities of the upgraded bioreactor platform and P3S3 scaffolds in creating more physiologically relevant *in vitro* models for bone biology research.

“Study carried out within the BIGMECH project - funded by European Union - Next Generation EU within the PRIN 2022 program (D.D. 104 - 02/02/2022 Ministero dell’Università e della Ricerca)”.

## 17. Do polystyrene nanoplastics affect the biological features of dog mesenchymal stromal cells *in-vitro*?

Paolo Di Lorenzo^1^, Martina Tambassi^2^, Melissa Berni^2^, Virna Conti^1^, Simona Bussolati^1^, Roberto Ramoni^1^, Erika Scaltriti^2^, Giuseppina Basini^1^, Stefano Grolli^1^

^1^Dipartimento di Scienze Medico-Veterinarie, Università di Parma, Parma, Italy. ^2^Risk Analysis and Genomic Epidemiology Unit, Istituto Zooprofilattico Sperimentale della Lombardia e dell’Emilia- Romagna, Parma, Italy.

Objective: Plastic pollution is one of the major environmental problems of our time. In particular, microplastics and nanoplastics pose a threat not only to the environment but also to living organisms, raising questions about their potential effects on health. The aim of this study was to investigate the possible biological effects of polystyrene nanoplastics (PS-NPs) on the function of canine adipose tissue-derived mesenchymal stromal cells (cMSCs) *in vitro*. cMSCs were chosen because of their well-documented regenerative potential and immunomodulatory properties, making them a valuable model for assessing the potential adverse effects of NP exposure. Domestic dogs are an excellent biomedical model as their close cohabitation with humans contributes to the development of comparable physio-pathological conditions.

Materials and methods: The effects of PS-NPs (~100 nm) ranging from 5 to 300 µg/mL on cMSCs were evaluated by assessing cytotoxicity and cell proliferation (MTT and BrdU assays), clonogenic capacity (CFU assay), adipogenic differentiation (Oil Red staining), and immunomodulation (IDO1 assay). NPs-cMSCs interaction was analyzed by microscopy using fluorescent PS-NPs. In addition, gene expression was investigated by RNA-Seq for a global transcriptomic profile, followed by real-time PCR targeting three functional genes of cMSCs: interleukin-8 (IL-8), vascular endothelial growth factor (VEGF), and superoxide dismutase 2 (SOD2).

Results: No significant effects were observed in adipogenic differentiation, IDO1 assay, clonogenic capacity and gene expression analysis. The MTT assay showed a slight increase in signal at 200 µg/mL (*P* < 0.001) compared to the untreated control and other NPs treatments. The BrdU assay showed a significant increase in DNA synthesis only at 300 µg/mL (*P* < 0.001). Fluorescence microscopy confirmed the interaction of cMSCs with PS-NPs and their internalization.

Conclusions: Our findings suggest that PS-NPs elicit minimal toxic effects on cMSCs. This contrasts with other *in vitro* studies that have reported toxic effects of nanoplastics on different cell models. This discrepancy may be due to several factors, including NPs type, size, the concentration used, and the duration of exposure. Further research is needed to elucidate the effects of NPs on cMSCs, given their significant physiological and therapeutic roles. Specifically, future studies should investigate the potential for inherent resistance mechanisms of cMSCs to these particles.

## 18. Treating keratoconjunctivitis sicca with canine mesenchymal stromal cells

Kristina Dojchinovska^1^, Caterina Morera^1^, Alessia Sulla^2^, Gabriele Scattini^1^, Luisa Pascucci^1^, Rolando Arcelli^1^

^1^Department of Veterinary Medicine, University of Perugia, Perugia, Italy. ^2^Department of Medicine and Surgery, University of Perugia, Perugia, Italy.

Objective: Keratoconjunctivitis sicca (KCS) is a relatively common disease in dogs characterized by dysfunction in tear production. As a result, the watery component of the tear film decreases or is absent, the eye becomes dry, and surface friction increases. This leads to inflammation and secondary infection of the cornea and conjunctiva with hyperemia and ocular discharge, often worsening over time, leading to changes in ocular surface integrity, increased corneal opacity, corneal defects, discomfort, pain, and eventually loss of vision. Among the various causes, the immune-mediated is the most frequent. Diagnosis is made by an ophthalmic examination and by assessing reflex tear production using the Schirmer tear test (STT). KCS is medically managed with the application of tear stimulants and replacements. However, in many cases, it is refractory to treatment and tear substitutes are required indefinitely. Mesenchymal stromal cells (MSCs) have been found to provide alleviation of clinical signs over long periods after the administration of a single dose. In some cases, tear production was increased or even restored to normal values.

Materials and methods: In this study, we assessed the therapeutic effects of canine adipose-derived MSCs as an alternative treatment for KCS in 4 dogs of different ages and breeds. All dogs had severe cases of KCS that were nonresponsive to previous treatments. Besides standard ophthalmic evaluation, an Optical Coherence Tomography (OCT) scan of the cornea was performed to assess the corneal integrity pre-treatment (day 0) and on day 14 post treatment. MSCs were transplanted in a dose of 2 × 10^6^ cells, administered as two injections per eye, one in the dorsal lacrimal gland and the other in the gland of the third eyelid.

Results: All dogs had an increase in STT values. The clinical appearance of the eyes improved, with reductions in ocular discharge and hyperemia, as well as decreased corneal opacity. The approach of using OCT scans to assess corneal integrity indicated that light could penetrate the cornea much better after 2 weeks post treatment, the endothelium became visible, and the surface of the cornea was lighter and more lucid.

Conclusions: The results obtained suggest an improvement in the condition for all 4 dogs; however, further studies with a larger number of patients, long-term follow-up, and the inclusion of cases ranging from mild to severe are needed to assess the long-term effects of MSCs.

## 19. Proteomic comparative analysis between equine amniotic mesenchymal stromal cells and their extracellular vesicles

Giulia Gaspari^1^, Alessio Soggiu^2^, Fausto Cremonesi^1^, Anna Lange-Consiglio^1^

^1^Department of Veterinary Medicine and Animal Science (DIVAS) Laboratory of Reproduction and Regenerative Medicine, Università degli Studi di Milano, Lodi, Italy. ^2^Dipartimento di Scienze Biomediche, Chirurgiche e Odontoiatriche, Università degli Studi di Milano, Milan, Italy.

Objective: A lot of evidence has highlighted that the therapeutic effects of mesenchymal stromal cells (MSCs) are, at least in part, mediated by the secretion of paracrine soluble functional factors and/or extracellular vesicles (EVs). The aim of this study was to compare specific functional features of equine amniotic mesenchymal cells (eAMCs) and their EVs through proteomic analysis.

Materials and methods: A pool of isolated eAMCs, obtained from three different amniotic membranes, was expanded until passage 3. eAMCs were characterized according to the International Society of Cell Therapy criteria, while the EVs (obtained by ultracentrifugation) were characterized in accordance with the 2024 Minimal Information for Studies of Extracellular Vesicles. On both eAMCs and EVs, a proteomic analysis was carried out. The raw data files were analyzed using FragPipe 22 and UniProtKB Equus caballus db (02.2025) to obtain protein identifications (FDR 0.01) and their respective label-free quantification values using recommended parameters. Statistical analysis was performed based on the combined_protein.tsv file on Fragpipe Analyst. Contaminant proteins were filtered out, and for the quantitation, proteins with < 2 peptides (and < 90% non-missing values) have been removed. A cut-off of the adjusted *P*-value of 0.05 along with a | log2 fold change| of 1 has been applied to determine differentially expressed proteins in the comparison.

Results: A total of 4,045 proteins were identified. Of these, 3,516 have been identified with more than two peptides. 2,323 are exclusive of eAMCs, 792 are in common between eAMCs and EVs, and 73 are exclusive of EVs. Several of the proteins present in EVs, but absent or reduced in eAMCs, are involved in biological processes related to tissue regeneration. The more significant ones are enzymes affecting inflammatory responses, such as Sulfhydryl oxidase, which modulates redox balance; proteins promoting tissue remodeling, like Periostin; Annexins, which reduce inflammatory mediator production through phospholipase A2 inhibition; Fibromodulin that can modulate inflammation resolution through TGF-β activity regulation; and other signaling molecules involved in immune modulation.

Conclusions: The presence of these proteins suggests that eAMCs likely exert their therapeutic effect primarily through EV secretion, positioning EVs as a promising resource for developing innovative cell-free therapeutic strategies in regenerative medicine.

## 20. Secretome and extracellular vesicle signatures in bone marrow-derived mesenchymal stromal cells after expansion in standard and next-generation media

Giulio Grieco, Simona Piccolo, Enrico Ragni, Laura de Girolamo

Laboratorio di Biotecnologie Applicate all’Ortopedia, IRCCS Ospedale Galeazzi - Sant’Ambrogio, Milano, Italy.

Objective: Mesenchymal stem cells (MSCs) are a promising therapeutic strategy for osteoarthritis (OA), largely due to their regenerative potential, which is attributed in part to their secretome, including soluble factors and extracellular vesicles (EVs). Since MSCs are sensitive to various culture conditions, this study aims to investigate the effects of different media supplemented with either FBS (F), platelet lysate (P), or serum/xeno-free (S/X) on the composition and therapeutic potential of the secretome from bone marrow-derived MSCs (BMSCs).

Materials and methods: BMSCs were cultured in F, P, or S/X media, with secretomes collected after starvation. The secretomes were analyzed for soluble factors, EVs, and miRNAs. Inflammatory responses were assessed in an *in vitro* OA model using inflamed chondrocytes and gene expression was evaluated by qRT-PCR.

Results: The secretomes from all conditions exhibited a similar molecular fingerprint. Proteomic analysis identified 98 common proteins encompassing growth factors and inflammatory mediators. EVs showed similar size and phenotype, with a slight difference in CD44 expression in EVs derived from platelet lysate-expanded MSCs. Despite high overall similarity, miRNA profiling identified 13 key players, with subtle differences in the miRNA composition of EVs from FBS-expanded BMSCs. All secretomes exhibited anti-inflammatory effects, with the FBS-expanded secretome showing the most pronounced therapeutic potential.

Conclusions: The secretomes derived from different culture conditions share key molecular components. EVs may contribute to variations in therapeutic outcomes through their cargo. Optimizing MSC expansion conditions is crucial for enhancing the therapeutic potential of MSC-derived secretomes in OA treatment. Further research is needed to clarify the specific role of factors, miRNAs, and EVs in modulating OA pathology.

## 21. From mesenchymal stem cells to bone: the role of biomechanical and electromagnetic stimulation in guiding msc differentiation

Ranveer Kaur^1^, Farah Daou^1^, Stefano Gabetti^2,3^, Beatrice Masante^2,3^, Eleonora Zenobi^4,5^, Carlotta Achille^5^, Elisa Scatena^4,5^, Simone Israel^2,3^, Cristina Bignardi^2,3^, Diana Massai^2,3^, Andrea Cochis^1^, Lia Rimondini^1^

^1^Department of Health Sciences, Center for Translational Research on Autoimmune and Allergic Diseases (CAAD), University of Eastern Piedmont (UPO), Novara, Italy. ^2^Department of Mechanical and Aerospace Engineering and PolitoBIOMed Lab, Politecnico di Torino, Turin, Italy. ^3^Interuniversity Centre for the Promotion of the 3Rs Principles in Teaching and Research, Turin, Italy. ^4^Hypatia Research Consortium, Rome, Italy. ^5^E. Amaldi Foundation, Rome, Italy.

Objective: Mesenchymal stem cells (MSCs) play a key role in bone regeneration, with their osteogenic differentiation guided by biochemical and biophysical cues. This study develops a 3D bone tissue model by seeding bone marrow-derived MSCs onto polylactic acid (PLA) scaffolds resembling trabecular bone in a perfusion bioreactor. Hydrostatic pressure from cyclic mechanical loading and pulsed electromagnetic field (PEMF) stimulation, mimicking physiotherapy, are applied.

Materials and methods: Scaffolds: 3D-printed PLA scaffolds (PLA600) were designed to replicate human trabecular bone and iliac crest morphology under physiological conditions. They provided structural support for bone marrow-derived mesenchymal stem cells (BMSCs) *in vitro*. Bioreactor: A custom bioreactor applied mechanical stimuli for bone tissue engineering, modulating fluid flow-induced shear stress via direct perfusion (0.006-24 mL/min). Cyclic hydrostatic pressure was introduced using a solenoid-actuated pinch valve with adjustable periods (0.25-30 s) and duty cycles (0%-100%). A commercial PEMF generator (1.5 mT, 75 Hz) enhanced osteogenic differentiation. Culture conditions: Constructs were cultured for 7 days under three conditions: (RP0) direct perfusion (flow rate of 0.3 mL/min) with intermittent hydrostatic pressure (24 h/day, 10 s period, 50% duty cycle, 5 kPa); (RP1) perfusion and intermittent hydrostatic pressure (2 h/day, 25 s period, 70% duty cycle, 13 kPa); and (RP1+PEMF) RP1 combined with PEMF stimulation (4 h/day) provided by IGEA, Italy.

Results: As confirmed by the metabolic assay, exposure to hydrostatic pressure and PEMF did not negatively affect cell viability. Cell distribution and morphology within the PLA600 scaffolds were further examined using live/dead staining and SEM analysis. However, the studies investigating their effect on osteogenic differentiation via gene expression analysis are still ongoing.

Conclusions: This study highlights the potential of combining biomechanical and electromagnetic stimuli with PLA600 scaffolds and a custom bioreactor platform to enhance MSC viability and bone regeneration and generate more physiologically relevant *in vitro* models for bone tissue engineering and regeneration.

“Study carried out within the BIGMECH project - funded by European Union - Next Generation EU within the PRIN 2022 program (DD 104 - 02/02/2022 Ministry of University and Research)”

## 22. The role of extracellular vesicles isolated from mesenchymal stromal cells in myofiber regeneration

Aurora Longhin^1^, Valentina Gatta^1^, Gabriella Teti^1^, Chiara Sassoli^2^, Flaminia Chellini^2^, Alessia Tani^2^, Martina Parigi^2^, Rachele Garella^3^, Francesco Palmieri^3^, Roberta Squecco^3^, Monica Mattioli Belmonte^4^, Caterina Licini^4^, Alessandra La Contana^4^, Sandra Zecchi-Orlandini^2^, Mirella Falconi^5^

^1^Department of Biomedical and Neuromotor Sciences, University of Bologna, Bologna, Italy. ^2^Department of Experimental and Clinical Medicine, section of Anatomy and Histology, University of Florence, Florence, Italy. ^3^Department of Experimental and Clinical Medicine, section of Physiology, University of Florence, Florence, Italy. ^4^Department of Clinical and Molecular Sciences, Università Politecnica delle Marche, Ancona, Italy. ^5^Department of Medical and Surgical Sciences, University of Bologna, Bologna, Italy.

Objective: Traumatic events can severely damage skeletal muscles, leading to serious consequences such as limited muscle regeneration, muscle loss, fibrosis, and impaired muscle function. While various therapeutic approaches, including cellular transplantation, have been explored to improve muscle regeneration, none have yielded satisfactory results so far. Cellular therapy utilizing mesenchymal stem cells (MSCs) shows great potential for muscle regeneration, particularly through the paracrine and immunomodulatory factors released by extracellular vesicles (EVs). Although the cellular and molecular mechanisms behind skeletal muscle regeneration are well-understood, the role of EVs in intercellular communication to coordinate the repair and regeneration of injured myofibers remains under investigation. Furthermore, the impact of EVs released by MSCs on myogenic repair and regeneration has not been thoroughly studied. Therefore, the goal of this study was to explore the role of EVs isolated from MSCs in muscle repair and regeneration.

Materials and methods: EVs were isolated from the culture medium of murine myoblasts using a liquid exchange method based on a polyethylene glycol precipitation protocol. The EVs derived from control, differentiated myoblasts, and MSCs were characterized for their size, membrane markers, and presence of myokines through electron microscopy, western blotting, and ProQuantum immunoassays. The EVs from MSCs were then added to the medium of damaged differentiated myoblasts to examine their effect on myofiber repair. The ability to produce myotubes was assessed through inverted light microscopy, and the expression of muscle differentiation markers, such as MyoD and myogenin, was evaluated through Western blotting.

Results: The results demonstrated that damaged myoblasts could release inflammatory factors encapsulated in EVs and that EVs isolated from MSCs played a role in reducing the inflammatory microenvironment, promoting myogenic repair, and aiding muscle regeneration.

Conclusions: Our findings highlight the significant role of MSC-derived EVs in regulating myogenic differentiation and regeneration following muscle damage.

This project has received funding from the Italian Ministry of University and Research, under the PRIN 2022 programme (20222P2NAJ; CUP I53D23003030006) (finanziato dall’Unione europea - Next Generation EU)

## 23. Low-intensity pulsed ultrasound as a promising tool for modulating cartilage regeneration

Cristina Manferdini^1^, Enrico Lenzi^1^, Elena Gabusi^1^, Diego Trucco^2,3^, Andrea Cafarelli^2,3^, Lorenzo Vannozzi^2,3^, Giovanni D’Atri^1^, Leonardo Ricotti^2,3^, Gina Lisignoli^1^

^1^Laboratorio di Immunoreumatologia e rigenerazione tissutale, IRCCS Istituto Ortopedico Rizzoli, Bologna, Italy. ^2^The BioRobotics Institute, Scuola Superiore Sant’Anna, Pisa, Italy. ^3^Department of Excellence in Robotics & AI, Scuola Superiore Sant’Anna, Pisa, Italy.

Objective: A major challenge in cartilage tissue engineering (TE) is the development of scaffolds capable of providing an instructive biomimetic environment to effectively drive MSC differentiation under both normal and inflammatory conditions. Hydrogels have emerged as promising biomaterials for this purpose, due to their biocompatibility and ability to mimic the tissue extracellular matrix. Graphene oxide (GO), known for its chondroinductive properties, and barium titanate piezoelectric nanoparticles (BTNPs), which can act as nanoscale transducers, are particularly promising nanomaterials for cartilage TE. Ultrasound waves also represent an intriguing approach to enhance chondrogenesis. However, the functions and mechanisms of low-intensity pulsed ultrasound (LIPUS) in osteoarthritis-related inflammation and regeneration remain unclear. The aim of this study was to evaluate LIPUS’ positive effect on cartilage formation by adipose-derived stem cells (ASCs) in a 3D hydrogel and in human osteoarthritic cartilage models.

Materials and methods: ASCs were embedded in a 3D VitroGel RGD® hydrogel, either without nanoparticles (Control) or doped with GO nanoflakes and BTNPs, and cultured under basal or inflammatory conditions (+IL1β 10 ng/mL). Cells were stimulated with defined LIPUS parameters (Ricotti *et al*., 2024) every 2 days until day 10 of culture, followed by chondrogenic differentiation for 28 days. Osteoarthritic cartilage explant tissues were also treated with LIPUS using the same parameters. At each time point, gene/ protein expression levels (SOX9, COL1A1, COL2A1, NFKB1, MMP13) and inflammatory cytokines release (IL6, CXCL8, TNF-α) were analyzed.

Results: LIPUS significantly promoted the chondrogenic differentiation of ASCs embedded in the 3D piezoelectric hydrogel, as evidenced by the overexpression of COL2 and ACAN and the reduced expression of the fibrotic marker COL1 compared to controls. LIPUS also exerted strong anti-inflammatory effects by downmodulating IL6, CXCL8, and TNF-α, while maintaining its ability to enhance chondrogenesis. Furthermore, cartilage explants treated with LIPUS exhibited a significant reduction in inflammatory cytokine levels and showed favorable modulation of chondrogenic transcriptional and degradative factors.

Conclusions: In conclusion, we demonstrated that LIPUS, as a non-invasive approach, represents a promising therapeutic strategy for counteracting inflammation and promoting cartilage regeneration. The use of controlled LIPUS stimulation may help bridge the gap between experimental research and future clinical treatments for osteoarthritis.

## 24. Mesenchymal stromal cells-derived extracellular vesicles as carriers for paclitaxel delivery

Angela Marcianti^1^, Eleonora Spampinato^1^, Sara Nava^1^, Giulia Maria Stella^2,3^, Simona Pogliani^1^, Simona Frigerio^1^, Mariangela Papa^1^, Fabio Moda^4,5^, Federico Angelo Cazzaniga^5^, Agelo Guido Corsico^2,3^, Catia Traversari^1^, Daniela Lisini^1^

^1^Cell Therapy Production Unit, Scientific Direction, IRCCS Neurologic Institute C. Besta Foundation, Milan, Italy. ^2^Department of Internal Medicine and Medical Therapeutics, University of Pavia Medical School, Pavia, Italy. ^3^Cardiothoracic and Vascular Department, Unit of Respiratory Diseases, IRCCS San Matteo Hospital Foundation, Pavia, Italy. ^4^Department of Medical Biotechnology and Translational Medicine, University of Milan, Milan, Italy. ^5^Unit of Laboratory Medicine, Laboratory of Clinical Pathology, IRCCS Neurologic Institute C. Besta Foundation, Milan, Italy.

Objective: Mesenchymal Stromal Cells-derived Extracellular Vesicles (MSC-EV) have emerged as a promising drug delivery system to improve anti-cancer therapies. We developed a GMP-compliant, reproducible, and standardized method for preparing MSC-EVs loaded with the chemotherapeutic drug Paclitaxel (PTX). We investigated their anti-proliferative activity, PTX content, and stability over time.

Materials and methods: EVs were obtained from adipose tissue-derived MSCs of 13 healthy donors via ultracentrifugation of culture supernatants. PTX-loaded EVs (EV-PTX) were prepared by adding PTX to the MSC culture medium prior to supernatant collection. EV identity was confirmed by nanoparticle tracking analysis (NTA) for concentration and size, transmission electron microscopy (TEM) for morphology, and surface marker expression analysis. We assessed their anti-proliferative activity against the mesothelioma cell line NCI H2052, PTX content using HPLC, and stability over time.

Results: From 80 mL of supernatants, we obtained an average of 7.03 ± 5.47 × 10^9^ total EV and 5.92 ± 2.92 × 10^9^ EV-PTX. The purified populations were homogeneous, with sizes ranging from 186 to 211 nm for EVs and 189 to 248 nm for EV-PTX. TEM confirmed the morphology and integrity of both EV and EV-PTX. Flow cytometry analysis showed high expression of EV markers (CD9, CD63 and CD81) and MSC markers (CD105, CD49e, CD146, CD44, and CD29), while platelet markers (CD41b, CD42a, and CD62p) were minimally expressed, indicating the absence of platelet lysate-derived EVs. EV-PTX exhibited significantly higher anti-proliferative activity against the NCI H2052 cell line compared to unmodified EVs, confirming their pharmacological efficacy despite the relatively low PTX content compared to EV-depleted supernatants. On average, 7.57 ± 2.79 × 10^9^ EV-PTX contained 0.54 ± 0.14 ng of PTX, whereas the PTX content in the supernatants was 36.18 ± 13.82 ng/mL. Stability studies demonstrated that EV and EV-PTX maintained their integrity and efficacy for up to 12 months when stored at -80°C in 0.9% NaCl supplemented with 1% DMSO.

Conclusions: These findings support the potential of EVs as drug delivery vehicles for anti-cancer therapy. Further studies are needed to optimize the EV-PTX dosage, improve loading efficacy, and evaluate the scalability of the EV preparation protocol to enable their clinical application in tumor treatment.

## 25. Differing responses of adipose-derived and bone marrow-derived mesenchymal stem cells during osteogenic differentiation

Elisa Mazzoni

Department of Chemical, Pharmaceutical and Agricultural Sciences, University of Ferrara, Ferrara, Italy.

Objective: With over 2 million bone grafts performed each year worldwide, bone regeneration is a primary task of tissue engineering. The use of human mesenchymal stem cells (hMSCs), alone or in combination with specific scaffolds and biomolecules, represents a promising therapy in this field. Several preclinical and clinical studies have investigated the use of hMSCs from bone marrow and adipose tissue to treat bone defects including non-union fractures, osteoarthritis, and pseudoarthrosis, but the results are often contradictory and incomplete. The aim of this study was to evaluate and compare the *in vitro* osteogenic and immunomodulatory properties of two cultures of MSCs derived from human bone marrow (hBMSCs) and from adipose tissue (hASCs).

Materials and methods: hBMSCs and hADSCs were seeded in 24-well plates and cultured with osteogenic medium up to day 40. To evaluate the osteogenic properties of stem cells, gene expression analysis was performed using RT 2 PCR Array technology at 14, 21, and 40 days of culture under osteogenic conditions and compared with cells grown on plastic vessel (TCPS). Immunomodulatory properties of MSCs were evaluated using the Bio-Plex Pro Human Cytokine 27-plex Assay on cell culture supernatants collected at days 3, 7, 14, and 21 under osteogenic conditions and compared with those from cells cultured on TCPS.

Results: Data obtained *in vitro* highlight that cultures of hBMSCs respond more efficiently to osteogenic induction compared to cultures of hASCs. Results from gene expression analysis showed an increased and more rapid expression of genes involved in the regulatory pathways of osteogenesis, in the regulation of extracellular matrix production, and in the transcriptional activation of other osteogenic genes in hBMSCs compared to hASCs. Furthermore, data from Bio-Plex Assay demonstrated that the components of the osteogenic medium exert an anti-inflammatory action on both stem cell cultures, reducing the protein expression of the main pro-inflammatory molecules in both hASCs and hBMSCs.

Conclusions: In conclusion, hBMSCs showed a greater osteogenic potential *in vitro* compared to hADSCs and the immunomodulatory properties appear to be very similar between the two cell cultures. Given the greater osteogenic properties and similar immunomodulatory effects of hBMSCs compared to hASCs, bone marrow appears to represent a better source of stem cells than adipose tissue for bone tissue regeneration.

## 26. Lyosecretome from mesenchymal stem cells for the treatment of bronchopulmonary dysplasia: production and biological activity *in vitro*

Angelo Modena^1^, Elia Bari^1^, Chiara Catozzi^2^, Claudia Moscheni^3^, Simona Turi^4^, Rossana Rossi^4^, Dario Di Silvestre^4^, Francesca Ricci^2^, Gino Villetti^2^, Maria Luisa Torre^1^

^1^Dipartimento di Scienze del Farmaco, Università del Piemonte Orientale, Novara, Italy. ^2^R&D Department, Chiesi Farmaceutici, Parma, Italy. ^3^Department of Biomedical and Clinical Sciences, University of Milan, Milano, Italy. ^4^Institute for Biomedical Technologies, National Research Council, Segrate, Italy.

Objective: This study investigates the preconditioning of mesenchymal stem cells (MSCs) with pro-inflammatory or hypoxic stimuli to produce a secretome with optimized biological activity for potential application in bronchopulmonary dysplasia.

Materials and methods: Secretome release from MSCs was induced by serum starvation for 48 h (Sser), serum starvation combined with IL-1β (10 ng/mL, IL-1β+Sser), or serum starvation under hypoxic conditions (5% O2, Hyp+Sser). The supernatants were collected, ultrafiltered (5 kDa) to isolate the secretome, and then freeze-dried using mannitol as a cryoprotectant. Lyosecretomes were characterized for particle size distribution (nanoparticle tracking analysis, NTA), morphology and ultrastructure (Transmission Electron Microscopy, TEM), proteomic profile, anti-elastase and anti-hyaluronidase activities, as well as cytocompatibility on human alveolar and bronchial epithelial cells.

Results: Pro-inflammatory and hypoxic preconditioning led to higher protein release from MSCs. Accordingly, after standardization based on protein content (22.5 mg/mg protein), higher yields were obtained for the IL-1β+Sser and Hyp+Sser conditions. MSCs also secreted lipids in varying amounts depending on the stimulus, with the highest lipid content observed in the Hyp+Sser batches. While freeze-drying did not affect extracellular vesicle (EV) particle size, MSC preconditioning did: the mean diameter, d50, and d90 values for IL-1β+Sser and Hyp+Sser batches were significantly larger than those of the Sser batches. After ultrafiltration and freeze-drying, EVs maintained their morphology and lipid bilayer without apparent differences among the different treatment groups. High anti-elastase activity was observed in the Sser and IL-1β+Sser batches at 20 mg/mL (~100%), whereas Hyp+Sser showed significantly lower activity (~80%). Anti-hyaluronidase assays confirmed higher activity for the SSer and IL-1β+Sser batches. Despite the lower enzymatic activity, proteomic analysis of the Hyp+Sser batches revealed increased expression of proteins related to oxidative stress response and anti-inflammatory pathways. Additionally, IL-1β+Sser and Hyp+Sser batches showed a positive impact on anti-fibrotic pathways compared to SSer. Finally, all secretome preparations demonstrated cytocompatibility at concentrations up to 3 mg/mL.

Conclusions: These findings highlight the influence of the MSC microenvironment on the composition, particle size, ultrastructure, and proteomic profile of EVs.

## 27. SDH-deficient gist modeling using genome-edited ipsc-derived mesenchymal stem cells

Ilaria Motta^1^, Livia Gozzellino^2^, Alice Costa^3^, Margherita Nannini^2,4^, Milena Urbini^5^, Maria Concetta Nigro^2,4^, Gianandrea Pasquinelli^2,6^, Maria Abbondanza Pantaleo^2,4^, Annalisa Astolfi^2,3^

^1^Alma Mater Institute on Healthy Planet, University of Bologna, Bologna, Italy. ^2^Department of Medical and Surgical Sciences (DIMEC), University of Bologna, Bologna, Italy. ^3^Department of Experimental, Diagnostic and Specialty Medicine (DIMES), IRCCS Azienda Ospedaliero-Universitaria di Bologna, Bologna, Italy. ^4^Division of Oncology, IRCCS Azienda Ospedaliero-Universitaria di Bologna, Bologna, Italy. ^5^Biosciences Laboratory, IRCCS Istituto Romagnolo per lo Studio dei Tumori (IRST) “Dino Amadori”, Meldola, Italy. ^6^Division of Pathology, IRCCS Azienda Ospedaliero-Universitaria di Bologna, Bologna, Italy.

Objective: Gastrointestinal stromal tumors (GISTs) are the most common type of mesenchymal tumor in the gastrointestinal tract, originating from interstitial cells of Cajal (ICCs) or their precursors. While most GISTs harbor mutations in KIT or PDGFRA, approximately 10%-15% lack these driver mutations. Among these, 20%-40% carry mutations in one of the four subunits of the succinate dehydrogenase (SDH) complex, with SDHA mutations being the most prevalent. SDH-deficient GISTs are typically located in the stomach, frequently metastasize to lymph nodes, and often exhibit slow progression even at advanced stages. The goal of this study was to characterize the gene expression profile of SDH-deficient GISTs and to develop a cellular model to investigate their biomolecular features and identify potential therapeutic targets.

Materials and methods: GIST samples were analyzed using gene expression arrays and RNA sequencing. In addition, induced pluripotent stem cells (iPSCs) carrying SDHA mutations were generated to create a cellular disease model. The SDHA mutation was introduced via CRISPR/Cas9-mediated genome editing, and its presence was confirmed by Sanger sequencing and western blot analysis of SDHB protein levels. The edited iPSCs were then differentiated into mesenchymal stromal cells. Successful differentiation was confirmed using real-time PCR and flow cytometry.

Results: SDH-deficient GISTs showed a distinct and homogeneous gene expression profile compared to KIT-mutant GISTs, characterized by elevated expression of neural markers and activation of hypoxia and fibroblast growth factor pathways. This molecular signature was used to validate an SDHA-knockout GIST cellular model derived from iPSCs. Differentiation into mesenchymal cells was confirmed by the loss of pluripotency markers (SOX2, OCT-4, NANOG) and the expression of mesenchymal markers (CD73, CD90, CD166). Furthermore, mesenchymal differentiation of SDHA-knockout iPSCs led to upregulation of neural markers (LHX2, N-CAM, NEFL) compared to wild-type cells.

Conclusions: This study defines the gene expression profile of SDH-deficient GISTs and demonstrates the successful establishment of an iPSC-based cellular model that recapitulates key disease features. Further research is required to fully characterize this model, which could serve as a valuable tool for preclinical pharmacological studies.

## 28. Study of the potential effects of different bioglass formulations on the cross-talk between ascs and hmecs

Clarissa Orrico^1^, Riccardo Pedraza^1^, Alessandro Mosca Balma^2^, Sara Meinardi^3^, Ilaria Roato^2^, Federico Mussano^2^

^1^Department of Mechanical and Aerospace Engineering, Politecnico of Turin, Turin, Italy. ^2^Department of Surgical Sciences, CIR Dental School, University of Turin, Turin, Italy. ^3^Department of Life Sciences and Systems Biology, University of Turin, Turin, Italy.

Objective: The development of a 3D-printed bone model that mimics the complex structure of bone could enable the investigation of interactions (cross-talk) between mesenchymal stem cells and endothelial cells, both of which play key roles in tissue regeneration. This study aims to analyze these interactions using a printed composite material designed to promote bone regeneration, including both vasculogenesis and bone formation.

Materials and methods: We used 2D-printed discs composed of a polycaprolactone (PCL) matrix, produced by solvent casting, either without additives or supplemented with 10% silica-based bioactive glass (SBA3), one variant of which was functionalized with copper (SBA3-Cu^2+^) to potentially enhance vasculogenesis. After sterilization, Human-Microvascular Endothelial Cells (H-MEC) and Adipose Tissue-Derived Stem Cells (ASCs) were seeded onto these three different matrices, either individually or in co-culture, using Endogrow medium for endothelial cell differentiation. Cell viability was assessed using a luminescent cell assay; adhesion and spreading were evaluated via immunofluorescence; osteogenic differentiation was analyzed by flow cytometry based on the expression of mesenchymal stem cell markers; osteocalcin (OCN) release was measured in culture supernatants using an immunoenzymatic assay; and (Ca^2+^) levels were quantified using a colorimetric assay.

Results: All matrices demonstrated good biocompatibility. ASCs cultured on PCL+SBA3 showed the highest viability immediately after seeding (day 1) as well as at days 5 and 7. In contrast, HMECs exhibited an initial adaptation phase but showed increased viability on PCL+SBA3 and PCL+SAB3-Cu^2+^ from day 3 onward. After 14 days, the percentage of CD105/CD90/CD73/ALP^+^ cells increased in co-cultures on PCL+SBA3 and PCL+SBA3-Cu^2+^ compared to PCL alone. Consistent with these findings, calcium release by ASCs was higher in the presence of bioglass after 21 days. OCN levels measured in culture supernatants after 28 days were highest in co-cultures on PCL+SBA3-Cu^2+^. Additionally, HMECs displayed a vasculature-like organization only when co-cultured with ASCs, regardless of the material used. Overall, these data suggest that bioglass at a 10% concentration primarily induces osteogenic differentiation rather than promoting vasculogenesis.

Conclusions: Endothelial cells and ASCs exhibited different organizational behaviors depending on the material composition. Our preliminary data indicate that 10% SBA3 bioglass promotes osteogenic differentiation of ASCs in co-cultures, while copper functionalization did not provide significant additional benefits.

## 29. The crosstalk between adipose-derived mesenchymal stem cells and microvascular endothelial cells in different culture conditions

Riccardo Pedraza^1^, Clarissa Orrico^1^, Alessandro Mosca Balma^2^, Sara Meinardi^3^, Ilaria Roato^2^, Federico Mussano^2^

^1^Department of Mechanical and Aerospace Engineering, Politecnico di Torino, Torino, Italy. ^2^Bone and Dental Bioengineering Laboratory, CIR Dental School, Department of Surgical Sciences, University of Torino, Torino, Italy. ^3^Department of Life Sciences and Systems Biology, University of Torino, Torino, Italy.

Objective: Mesenchymal stem cells (MSCs) can interact with endothelial cells to promote angiogenesis and tissue regeneration. This study aimed to investigate how co-culturing adipose-derived MSCs (ASCs) and human microvascular endothelial cells (HMECs) in three different culture media - αMEM (MSC expansion medium), MCDB131 (endothelial cell expansion medium), and Endogrow (angiogenesis differentiation medium) - affects osteogenic differentiation and vascularization.

Materials and methods: Co-cultures were established at an ASC:HMEC ratio of 1:3. Flow cytometry was used to assess the expression of surface markers characteristic of endothelial cells (CD31+), mesenchymal cells (CD105/CD73/CD90+), and osteo-committed mesenchymal cells (CD105/CD73/CD90/ALP+). Immunofluorescence microscopy was performed to visualize cell morphology and organization under different culture conditions.

Results: After 7 days, comparable growth of endothelial and MSCs was observed in αMEM and MCDB131, whereas Endogrow induced different behavior, likely due to its differentiation-promoting components. At 14 days, the proportion of osteo-committed mesenchymal cells increased in all conditions, while endothelial cell numbers decreased compared to day 7. Meanwhile, the population of undifferentiated mesenchymal cells exhibited a decreasing trend over time, suggesting a shift toward osteogenic differentiation. The direct crosstalk between ASCs and HMECs appeared to naturally regulate these cellular subsets, while the different media influenced their interactions, affecting lineage commitment and functional phenotype. Furthermore, the increase in osteo-committed cells was consistent with immunofluorescence images, which showed a predominance of ASCs despite their low initiating seeding ratio.

Conclusions: These findings highlight the crucial role of microenvironmental factors in directing ASC/HMEC behavior and provide valuable insights for optimizing culture conditions in regenerative applications.

## 30. Design and characterisation of a 3D *in vitro* model using human mesenchymal stromal cells for skin wound healing

Federica Re^1^, Luciana Sartore^2^, Elisa Borsani^1^, Chiara Pasini^2^, Federica Trenta^1^, Rosalba Monica Ferraro^3^, Camillo Almici^4^, Andrea Bianchetti^4^, Domenico Russo^1^

^1^Department of Clinical and Experimental Sciences, University of Brescia, Brescia, Italy. ^2^Department of Mechanical and Industrial Engineering, University of Brescia, Brescia, Italy. ^3^Department of Molecular and Translational Medicine University of Brescia, Brescia, Italy. ^4^Department of Transfusion Medicine, ASST Spedali Civili, Brescia, Italy.

Objective: Recent advancements in regenerative medicine have focused on developing innovative scaffolds for the treatment of skin injuries. This study evaluates the biocompatibility of gelatin-polyethylene glycol-chitosan-glycerol (G-PEG-CH-Gly) and gelatin-polyethylene glycol-glycerol (G-PEG-Gly) hydrogels with mesenchymal stromal cells (MSCs) derived from bone marrow (BM-MSCs) and umbilical cord (UC-MSCs), in the context of skin regeneration.

Materials and methods: The scaffolds’ morphology, chemical properties, and hydrolytic degradation were assessed over 25 days. Differentiation protocols for BM-MSCs and UC-MSCs toward fibroblasts and keratinocytes were developed and optimized using either fetal bovine serum (FBS) or human platelet lysate (HPL), in both 2D and 3D culture systems. Biocompatibility tests were conducted at 21 and 28 days. Differentiation markers for fibroblasts (COLL1, COLL4) and keratinocytes (p63, KRT10) were analyzed using QuantStudio Absolute Q Digital PCR. Histomorphological assessments were performed using hematoxylin-eosin and Masson-Goldner trichrome staining. Immunohistochemistry for COLL1 and KRT10 was carried out on the 3D models, with MatriDerm® serving as a control.

Results: Swelling ratio and mass loss tests demonstrated that the hydrogels maintained their structural integrity over time, and cell viability was sustained under all tested conditions. However, BM-MSCs and UC-MSCs exhibited different growth patterns within the scaffolds. In 2D culture, UC-MSCs exhibited significantly higher COLL1 expression under the FI HPL condition compared to BM-MSCs. COLL4expression was significantly elevated in both UC-MSCs and BM-MSCs when cultured with FI HPL. An increase in KRT10 expression was observed in BM-MSCs under KI HPL conditions. Compared to 2D cultures and MatriDerm®, cells cultured in G-PEG-CH hydrogels showed significantly higher expression of COLL1, COLL4, p63, and KRT10. Furthermore, UC-MSCs demonstrated superior differentiation toward the fibroblastic lineage in G-PEG-CH compared to MatriDerm®.

Conclusions: The G-PEG-CH-Gly and G-PEG-Gly hydrogels were biocompatible and supported MSC differentiation into fibroblasts and keratinocytes. Notably, UC-MSCs cultured in G-PEG-CH exhibited enhanced differentiation capacity, with elevated expression of KRT10 and p63 in the presence of HPL.

## 31. Human plasma creates a physiologic environment to select stem cells from bone marrow stromal cells

Alessia Repetto^1^, Anita Muraglia^1^, Ranieri Cancedda^2^, Gilberto Filaci^1,3^, Maddalena Mastrogiacomo^1,3^

^1^Dipartimento di Medicina Interna e Specialità Mediche, Università degli studi di Genova, Genova, Italy. ^2^Dipartimento di Medicina Sperimentale, Università degli studi di Genova, Genova, Italy. ^3^IRCCS Ospedale Policlinico San Martino, Genova, Italy.

Objective: Mesenchymal stem cells (MSCs) are widely used in regenerative medicine due to their remarkable differentiation potential. Adult tissue-derived stem cells share many characteristics with embryonic stem cells, and current research aims to establish a hierarchy between these two cell populations based on their properties. Culture conditions significantly influence the quality of MSCs and must be carefully controlled to preserve their functional features. In the search for more physiological culture systems for clinical applications, human plasma stands out as a natural environment in which cells operate. Platelet lysate (PL), a mixture of platelet-derived factors, and Fibroblast Growth Factor 2 (FGF-2) have both been used to modulate MSC stemness and lineage commitment. In this context, our study aimed to explore the role of a human plasma-supplemented medium, enriched with either FGF-2 or PL, in supporting the selection, maintenance of stemness, and proliferation of bone marrow-derived MSCs (BM-MSCs).

Materials and methods: BM-MSCs were cultured in media containing either 5% plasma and 1% PL (P+PL) or 5% plasma and FGF-2 (1 ng/mL) (P+F). At passages 1 and 3, both cell populations were analyzed for clonogenic potential (CFU-f assay), proliferation capacity (cumulative population doublings), and gene expression of OCT4 and BMP-2 as markers of stemness and lineage commitment, respectively (quantitative PCR). Additionally, MSC surface markers and Stage-Specific Embryonic Antigen 3 (SSEA-3), a pluripotency marker, were evaluated by flow cytometry.

Results: MSCs cultured in the P+PL condition exhibited a higher proliferation rate and greater clonogenic capability, whereas cells in the P+F condition showed slower growth and reduced colony formation. However, P+F-derived cells displayed higher expression of the stemness marker OCT4 and lower expression of BMP-2 compared to those in the P+PL condition. Flow cytometry analysis revealed a slightly higher proportion of SSEA-3-positive cells in the P+F condition than in the P+PL condition.

Conclusions: This study demonstrates that plasma supports cell growth only when supplemented with FGF-2 or PL. Cells derived from P+F media exhibited lower proliferative capacity but included a small subpopulation positive for CD105, CD90, and the pluripotency marker SSEA-3 - a subpopulation that was less prevalent in the PL condition, which favored a more proliferative cell population. These preliminary findings suggest that plasma combined with FGF-2 selectively enriches for cells with superior stemness properties, whereas PL promotes the expansion of more differentiated transit-amplifying cells.

## 32. Genome analysis of healthy dental pulp stem cells and periodontal ligament stem cells

Ilaria Roato^1^, Clarissa Orrico^2^, Riccardo Pedraza^2^, Alessandro Mosca Balma^1^, Giacomo Baima^1^, Mario Aimetti^1^, Federico Mussano^1^

^1^Dental School, Surgical Sciences Dep, University of Turin, Torino, Italy. ^2^Department of Mechanical and Aerospace Engineering, Politecnico di Torino, Torino, Italy.

Objective: Periodontal ligament stem cells (PDLSCs) and dental pulp stem cells (DPSCs) are mesenchymal stem cells (MSCs) that demonstrate multilineage differentiation potential *in vitro* and have been studied for their potential application in dental regenerative treatment. PDLSCs and DPSCs originate from distinct anatomical compartments within the tooth, and therefore, their genomic profiles are expected to differ; however, few data are currently available. In this study, we investigated the differentially expressed genes (DEGs) between healthy PDLSCs and DPSCs derived from a single healthy tooth.

Materials and methods: Total RNA was extracted from primary PDLSCs and DPSCs. RNA libraries were prepared using the Stranded mRNA Prep Kit and sequenced on the Illumina Novaseq X Plus platform. Pre-processing of sequencing reads was performed using fastp v0.20.0. A high percentage of uniquely mapped reads was achieved, with a mean value of 89%. Filtered reads were then mapped to the Homo sapiens reference genome using STAR v2.7.9a with standard parameters.

Results: We identified 1,905 statistically significant DEGs in DPSCs compared with PDLSCs. These genes were used for pathway annotation analysis in the Reactome database and for Gene Ontology (GO) analysis. Pathway enrichment analysis revealed differential expression in DPSCs versus PDLSCs in pathways such as extracellular matrix organization, neuronal system, signaling by interleukins, extracellular matrix degradation, and regulation of insulin-like growth factor (IGF) transport by IGF-binding proteins (IGFBPs). Functional enrichment analysis based on GO was conducted in three categories: molecular function, biological process, and cellular component. The top five molecular functions identified were: extracellular matrix binding, extracellular matrix structural constituent, extracellular matrix structural constituent conferring tensile strength, growth factor binding, and integrin binding. The top five biological processes were: external encapsulating structure organization, extracellular matrix organization, extracellular structure organization, kidney development, and renal system development. The top five cellular components were: collagen-containing extracellular matrix, complex of collagen trimers, endoplasmic reticulum lumen, membrane microdomain, and membrane raft.

Conclusions: These preliminary data reveal substantial differences between DPSCs and PDLSCs, suggesting that their distinct anatomical origins influence their phenotypes and behaviors. Therefore, careful consideration is needed when selecting these cells for regenerative applications.

## 33. Effect of conditioned medium from human amniotic mesenchymal stromal cells on inflammasome activation in M1 macrophages

Marta Rossi^1,2^, Marta Magatti^2^, Pietro Romele^2^, Elsa Vertua^2^, Elisabetta Giuzzi^2^, Alice Paini^2^, Anna Pasotti^2^, Silvia De Munari^2^, Antonietta Rosa Silini^2^, Ornella Parolini^1,3^

^1^Department of Life Science and Public Health, Università Cattolica del Sacro Cuore, Rome, Italy. ^2^Centro di Ricerca E. Menni, Fondazione Poliambulanza Istituto Ospedaliero, Brescia, Italy. ^3^Fondazione Policlinico Universitario “Agostino Gemelli” IRCCS, Rome, Italy.

Objective: Our previous studies have shown that conditioned medium derived from human amniotic membrane mesenchymal stromal cells (CM-hAMSC) significantly modulates myeloid cells by reducing the pro-inflammatory M1 macrophage phenotype and promoting the anti-inflammatory M2 phenotype. Macrophages play a central role in inflammation, acting as key regulators and effectors of the immune response. In response to harmful stimuli such as lipopolysaccharides (LPS), macrophages activate inflammasomes, especially the NOD-like receptor family pyrin domain-containing 3 (NLRP3) inflammasome. NLRP3 activation drives M1 polarization, leading to metabolic reprogramming, production of reactive oxygen species (ROS), and secretion of pro-inflammatory cytokines. Canonical activation of the NLRP3 inflammasome requires oligomerization of the apoptosis-associated speck-like protein containing a caspase activation and recruitment domain [CARD] (ASC) with NLRP3 and caspase-1, resulting in the production of IL-1β and IL-18 and inducing pyroptotic cell death. Given the critical role of NLRP3 inflammasome activation in M1 polarization, this study investigates whether CM-hAMSC can modulate LPS-induced inflammasome activation, potentially offering insights into its immunomodulatory effects.

Materials and methods: M1 macrophages were generated *in vitro* by culturing monocytes with GM-CSF for five days. Inflammasome activation was induced by stimulating these macrophages with LPS and adenosine triphosphate (ATP), in the presence or absence of CM-hAMSC. Five hours after treatment, inflammasome activation was assessed by evaluating ASC speck formation, IL-1β production, and mitochondrial ROS accumulation. These parameters were analyzed using fluorescence microscopy, ELISA, and flow cytometry.

Results: Preliminary findings indicate that CM-hAMSC inhibits inflammasome activation by reducing IL-1β release and ROS accumulation in macrophages. However, CM-hAMSC does not appear to affect ASC speck formation, suggesting that the reduction in IL-1βoccurs downstream of the inflammasome assembly. Further investigations are required to clarify the underlying mechanisms.

Conclusions: This study demonstrates that CM-hAMSC suppresses inflammasome activation in M1 macrophages, highlighting its anti-inflammatory potential. Further research is needed to fully elucidate the molecular pathways by which CM-hAMSC modulates inflammasome activation in macrophages.

## 34. Inward rectifier and calcium-activated potassium currents in amniotic fluid-derived cells

Paola Sabbatini^1^, Sabrina Cipriani^2^, Andrea Biagini^1,3^, Luana Sallicandro^1,3^, Flora Ballarino^1^, Cataldo Arcuri^3^, Rita Romani^3^, Paolo Prontera^3^, Alessandra Mirarchi^3^, Rosaria Gentile^1,4^, Diletta Del Bianco^1^, Elko Gliozheni^1,3,5^, Sandro Gerli^3,4,6^, Irene Giardina^3,4,6^, Maurizio Arduini^3,4,6^, Alessandro Favilli^3,4,6^, Antonio Malvasi^7^, Andrea Tinelli^8^, Bernard Fioretti^1,4^

^1^Department of Chemistry, Biology and Biotechnologies, University of Perugia, Perugia, Italy. ^2^Rheumatology Unit, Department of Medicine, School of Medicine, University of Perugia, Perugia, Italy. ^3^Department of Medicine and Surgery, University of Perugia, Perugia, Italy. ^4^Laboratorio Interdipartimentale di Fisiopatologia della Riproduzione, Università degli Studi di Perugia, Perugia, Italy. ^5^Department of Obstetrics and Gynecology, Faculty of Medicine, University of Tirana, Tirana, Albania. ^6^Centre of Perinatal and Reproductive Medicine, Department of Obstetrics and Gynecology, University of Perugia, Perugia, Italy. ^7^Department of Biomedical Sciences and Human Oncology, University of Bari, Bari, Italy. ^8^Department of Obstetrics and Gynecology and CERICSAL (CEntro di RIcerca Clinico SALentino), Veris delli Ponti Hospital, Scorrano, Italy.

Objective: Human amniotic fluid (AF) is a dynamic and complex biological environment that surrounds the developing fetus during pregnancy. It contains a heterogeneous population of stem cells originating from embryonic and extra-embryonic tissues, which possess significant therapeutic and regenerative potential. Among these, amniotic fluid stem cells (AF-SCs) can be isolated and have demonstrated notable neurogenic potential. *In vitro* studies have shown that, under the influence of specific growth factors and molecular signals that mimic the neural environment, AF-SCs can be induced to differentiate into both neuronal and glial cells. Additionally, AF-SCs are capable of secreting neurotrophic factors, which support the growth, survival, and differentiation of neurons. These factors can positively influence the microenvironment of damaged neural tissue, facilitating repair and regeneration. When cultured with basic fibroblast growth factor (bFGF), AF-SCs have shown the ability to survive and migrate following transplantation into the striatum of rat brains, exhibiting characteristics of neuronal and glial progenitor cells.

Materials and methods: In this study, we conducted an electrophysiological analysis to investigate the differentiation of AF-SCs into glial and neuronal cells in the presence of bFGF.

Results: Using patch-clamp techniques, we observed a fibroblast-like morphology, along with barium-sensitive inward-rectifying potassium currents (Kir) and calcium-activated potassium currents (KCa). We also examined the electrophysiological properties and calcium dynamics in response to histamine, a marker of undifferentiated neural progenitors. Histamine was found to elevate intracellular calcium levels, as measured using Fura-2, and to activate KCa currents, showing a temporal profile similar to that observed in AF-SCs.

Conclusions: These findings confirm the presence of both Kir and KCa currents in AF-SCs, with KCa currents being regulated by endogenous stimuli such as histamine.

## 35. Development of a potency assay to evaluate the immunomodulatory potential of canine mesenchymal stromal cells

Gabriele Scattini^1^, Martina Pellegrini^2^, Alessia Sulla^3^, Giulio Severi^2^, Kristina Dojchinovska^1^, Monica Cagiola^2^, Luisa Pascucci^1^

^1^Department of Veterinary Medicine, University of Perugia, Perugia, Italy. ^2^Istituto Zooprofilattico Sperimentale dell’Umbria e delle Marche “Togo Rosati”, Perugia, Italy. ^3^Department of Medicine and Surgery, University of Perugia, Perugia, Italy.

Objective: Mesenchymal stromal cells (MSCs) are tissue-derived cells that play a key role in modulating inflammation. Since their discovery in the 1960s, MSCs have been widely studied. Originally referred to as “stem cells”, their classification has been revised several times. MSCs possess immunomodulatory properties and therapeutic potential for the treatment of various conditions, including immune-mediated and chronic degenerative diseases. However, characterization based solely on differentiation capacity, immunophenotype, plastic adherence, and colony formation does not fully define their origin, identity, and functions. Moreover, there is an urgent need to develop protocols capable of assessing and predicting their efficacy in terms of angiogenic, anti-inflammatory, and immunomodulatory activities, especially as their application in regenerative medicine expands in both humans and animals. In this study, we investigated the ability of MSCs isolated from canine adipose tissue to modulate the immune response by evaluating their *in vitro* effects on DH82 cells, a canine macrophage-like cell line.

Materials and methods: Tumor necrosis factor alpha (TNFα) was used as an indicator to assess MSC efficacy. TNFα levels in the culture supernatant were measured using a bioassay based on the sensitivity of L929 cells to this cytokine. It has been demonstrated that TNFα induces either necrosis or apoptosis in L929 cells. DH82 cells were seeded alone or co-cultured with MSCs at various ratios and then stimulated with 100 ng/mL lipopolysaccharide (LPS). Supernatants were collected at regular intervals for analysis. After 72 h, cells were analyzed by flow cytometry and immunofluorescence to assess activation status relative to cell ratios. TNFα concentrations in the collected supernatants were measured using the L929 cell-based bioassay, as L929 cells exhibit a dose-dependent response to TNFα. By analyzing residual viability using an MTT assay, TNFα concentrations could be accurately quantified.

Results: Preliminary results indicated that TNFα levels produced by LPS-stimulated DH82 cells were significantly reduced in the presence of MSCs.

Conclusions: The protocol developed in this study enables the time-dependent analysis of a large number of samples at relatively low costs, facilitating scalable screening processes for donor selection and MSC evaluation.

## 36. Engineered extracellular vesicles from mesenchymal stromal cells as nano-shuttles for viral angiogenic oligopeptides

Gabriele Scattini^1^, Giulia Venneri^2^, Cinzia Giagulli^2^, Giulio Alessandri^3^, Luisa Pascucci^1^

^1^Department of Veterinary Medicine, University of Perugia, Perugia, Italy. ^2^Department of Molecular and Translational Medicine, University of Brescia, Italy. ^3^Image Clinic, Milan, Italy.

Objective: Inadequate angiogenesis contributes to several diseases and impaired healing processes. To promote tissue reperfusion, the administration of proteins or oligopeptides with angiogenic properties offers a promising therapeutic approach. However, the unfavorable pharmacokinetic properties of proteins and oligopeptides often limit their clinical application. Extracellular vesicles (EVs) represent natural delivery systems due to their inherent biological features. Mesenchymal stromal cell-derived EVs (MSC-EVs) have been explored as drug carriers, leveraging their ability to target injured tissues. In this study, we investigated the angiogenic potential of oligopeptides (OPs) derived from the HIV-1 matrix protein p17. Human and equine MSCs were engineered to produce EVs decorated with p17-derived OPs. To this end, OP sequences were fused to CD63, a tetraspanin protein naturally enriched in EVs, and the green fluorescent protein (GFP) sequence was included as a reporter.

Materials and methods: The angiogenic potential of p17-derived OPs was assessed *in vitro* using human and equine endothelial cells. Tube formation, wound healing, and proliferation assays were performed. Sequences encoding angiogenic p17-derived OPs were successfully cloned into a vector expressing CD63-GFP. Human MSCs and equine adipose-derived MSCs were then transfected to express OPs on the EV membrane. EVs were isolated from culture media by ultrafiltration and characterized by nanoparticle tracking analysis (NTA), electron microscopy (EM), and Western blotting (WB).

Results: Engineered EVs displayed typical dimensions and morphology. EM and NTA analyses revealed size distributions ranging from 70 to 500 nm, with the size peak varying among different OPs. Western blot analysis confirmed the presence of GFP and typical EV markers in the enriched vesicle preparations.

Conclusions: These preliminary findings show that the CD63-mediated EV engineering strategy is effective for producing functionalized EVs. However, further optimization is required to improve transfection efficiency and vesicle yield. Additional *in vitro* studies on endothelial cells are needed to confirm whether the engineered EVs retain the angiogenic activity of p17-derived OPs.

FUNDING - PRIN 2022 - Finanziato dall’Unione Europea - Next Generation EU - ID 2022BJB5FP.

## 37. *In vitro* comparison of immunomodulatory properties of mesenchymal stromal cells from human term placenta

Alessandra Spanò^1^, Elsa Vertua^2^, Pietro Romele^2^, Patrizia Bonassi Signoroni^2^, Antonietta Rosa Silini^2^, Ornella Parolini^1,3^

^1^Fondazione Policlinico Universitario “Agostino Gemelli” IRCCS, Rome, Italy. ^2^Centro di Ricerca E. Menni, Fondazione Poliambulanza Istituto Ospedaliero, Brescia, Italy. ^3^Department of Life Science and Public Health, Università Cattolica del Sacro Cuore, Rome, Italy.

Objective: Mesenchymal stromal cells (MSCs) have revolutionized regenerative medicine due to their unique properties, particularly their multipotency and potent immunomodulatory capabilities. MSCs can be isolated from adult tissues, such as bone marrow and adipose tissue, as well as from perinatal tissues, including fetal membranes and the umbilical cord (e.g., Wharton’s jelly). Perinatal tissues offer several advantages: they are readily available as biological waste, and MSCs from these sources are easy to isolate and expand. Moreover, increasing evidence suggests that MSC secretomes exhibit functional properties similar to those of their parent cells. However, from a clinical perspective, there is no definitive evidence demonstrating the superiority of secretomes from any particular source. Therefore, comprehensive characterization of tissue-specific MSC secretomes is essential for identifying the most effective therapeutic options, especially for disease-specific applications. Given that immunomodulation is a critical mechanism through which MSC secretomes promote tissue regeneration, this study aims to compare the immunomodulatory properties of secretomes derived from MSCs isolated from the amniotic membrane (hAMSCs) and from Wharton’s jelly of the umbilical cord (hWJ-MSCs).

Materials and methods: Human amniotic membrane-derived MSCs (hAMSCs) were isolated using enzymatic digestion following standard protocols. Wharton’s jelly-derived MSCs (WJ-MSCs) were obtained via the explant culture method. Secretomes (i.e., conditioned media) were collected after seeding MSCs in 24-well plates and culturing them for five days at 37 °C. The immunomodulatory properties of the secretomes were assessed by evaluating their effects on T cell activation and proliferation, T helper cell polarization, regulatory T cell induction, and macrophage polarization, specifically the shift from the pro-inflammatory M1 phenotype to the anti-inflammatory M2 phenotype.

Results: Preliminary studies indicate that hAMSCs and WJ-MSCs exhibit comparable abilities to modulate both innate and adaptive immune cells. Ongoing analyses are being conducted to confirm these findings.

Conclusions: Further investigations will expand this comparison to include MSCs derived from additional perinatal tissues, such as amniotic fluid-derived stem cells.

## 38. 2.5D analysis of 3D spheroids for cancer stem cell isolation

Mariachiara Stellato^1^, Vágó Pal^2^, Akos Diosdi^2,3^, Maria Harmati^2^, Daniel Remondini^1^, Nicola Normanno^4^, Gastone Castellani^5,6^, Filippo Piccinini^4,5^, Peter Horvath^2,3^

^1^Department of Physics and Astronomy “Augusto Righi” (DIFA), University of Bologna, Bologna, Italy. ^2^Synthetic and System Biology Unit, HUN-REN Biological Research Centre (BRC), Szeged, Hungary. ^3^Single-Cell Technologies Ltd, Szeged, Hungary. ^4^IRCCS Istituto Romagnolo per lo Studio dei Tumori (IRST) “Dino Amadori”, Meldola (FC), Italy. ^5^Department of Medical and Surgical Sciences (DIMEC), University of Bologna, Bologna, Italy. ^6^IRCCS Azienda Ospedaliero-Universitaria di Bologna S.Orsola, Bologna, Italy.

Objective: Cancer stem cells (CSCs) have attracted significant attention in cancer research due to their unique traits, including enhanced resistance to conventional anti-cancer drugs and therapies. To better understand their role and develop strategies to overcome their drug resistance, CSCs can be studied in controlled environments such as 3D multicellular spheroids. Notably, interactions between CSCs and mesenchymal stem cells (MSCs) have been shown to influence tumor development. Isolating single cells from CSC-MSC co-cultures can provide valuable insights into their behavior and mechanisms of drug resistance.

Materials and methods: To isolate and study single cells from 3D aggregates, we employed a sectioning protocol that reduces the culture into a sequence of 2D slices. These thin sections can then be analyzed to identify and collect cells of interest using laser-based techniques and transferred into an Eppendorf tube. Although promising and informative, this method has a major limitation: sectioning spheroids with a microtome or cryostat inevitably damages the culture, compromising the ability to perform reliable studies on the morpho-biological properties of selected cells. Based on this understanding, we developed an optimized protocol for single-cell extraction from spheroids by adjusting the section thickness to preserve cell morphology. This innovation enables the possibility of conducting radiomics studies on individual cells within aggregates.

Results: We conducted several experiments to evaluate this new protocol using spheroids of various sizes and cell line combinations, including mesenchymal stem cells and cancer cells. The results showed that it is possible to successfully retrieve single cells from different regions of the spheroids without damaging the selected cells.

Conclusions: This method represents an initial step toward isolating individual cells without enzymatic digestion. A potential future development could involve creating a system of micro-pipettes capable of penetrating spheroids and retrieving selected cells directly, eliminating the need for sectioning.

## 39. Validation of cryopreserving media for canine mesenchymal stromal cells: a further step toward therapy

Alessia Sulla^1^, Gabriele Scattini^2^, Luisa Pascucci^2^, Ida Duprez^3^, Gianluca Moretti^4^

^1^Department of Medicine and Surgery, University of Perugia, Perugia, Italy. ^2^Department of Veterinary Medicine, University of Perugia, Perugia, Italy. ^3^StromaBio AB, Solna, Sweden. ^4^IGA Technology Services, Parco Scientifico e Tecnologico “L. Danieli”, Udine, Italy.

Objective: Despite the growing interest in cell-based therapies in veterinary medicine, few studies have explored cryopreservation media suitable for clinical application. The requirement to remove dimethyl sulfoxide (DMSO) and fetal bovine serum (FBS) before administration limits the practicality of MSC-based treatments. Recently, ready-to-use commercial cryomedia (CM) that are free of xenogeneic components and contain no or reduced DMSO have become available. By eliminating the need for extensive washing steps after thawing, these media could streamline workflows, save time in clinical settings, and enhance reproducibility in research. In the current study, five pre-formulated CMs were tested and compared with the standard in-house medium commonly used for MSC cryopreservation. The aim was to identify an FBS-free cryopreservation medium that is free of DMSO or has a reduced DMSO concentration, while maintaining cell viability and metabolic function.

Materials and methods: MSCs isolated from the adipose tissue of three dogs were cryopreserved at passage 3 using the following cryomedia:

· CryoStor® CS10 (STEMCELL Technologies): animal component-free, 10% DMSO. · CryoStor® CS5 (STEMCELL Technologies): animal component-free, 5% DMSO. · 2-8CELLsius^TM^ 10 (Protide Pharmaceuticals): protein-free, animal component-free, 10 % DMSO. · 2-8CELLsius^TM^ 5 (Protide Pharmaceuticals): protein-free, animal component-free, 5% DMSO. · PRIME XV Stem FreezIS (Fujifilm Irvine Scientific): DMSO-free, protein-free, animal component-free. · CONTROL MEDIUM: 90% FBS, 10% DMSO.

Cryopreservation effectiveness was evaluated based on three main criteria after thawing: recovery rate of total and viable cells, cell recovery at 24 and 72 h, and cellular metabolic activity.

Results: PRIME XV, a DMSO- and animal protein-free cryomedium, demonstrated comparable or superior performance to DMSO-containing solutions in maintaining MSC viability and function. In contrast, 2-8CELLsius cryomedia exhibited the lowest effectiveness among the tested formulations. Notably, after 72 h of reseeding, MSCs cryopreserved with PRIME XV showed the highest doubling rate, which correlated with a significant increase in metabolic activity at 24 h post-thaw.

Conclusions: Our findings highlight the potential of PRIME XV as a ready-to-use alternative to conventional CM, eliminating xenogeneic and toxic components. This advancement could enhance the accessibility and clinical applicability of regenerative medicine for veterinary patients.

## 40. Donor sites and harvesting techniques affect mirna cargos of extracellular vesicles releasedby human adipose-derived mesenchymal stromal cells

Michela Maria Taiana, Caterina Visconte, Alessandra Colombini, Paola De Luca, Enrico Ragni, Laura de Girolamo

Laboratorio di Biotecnologie Applicate all’Ortopedia, IRCCS Ospedale Galeazzi - Sant’Ambrogio, Milan, Italy.

Objective: Osteoarthritis (OA) is a degenerative joint disorder characterized by cartilage deterioration, pain, and functional impairment, driven by inflammatory and catabolic processes. Adipose-derived stem cells (ASCs) have emerged as a promising therapeutic strategy due to their regenerative potential, which is primarily mediated by extracellular vesicles (EVs) carrying bioactive molecules, including microRNAs (miRNAs). These miRNAs can modulate target cell functions through gene expression regulation. This study aimed to investigate the effects of adipose tissue collection site (abdominal *vs*. peritrochanteric) and harvesting technique (surgical excision *vs*. lipoaspiration) on the miRNA profile of ASC-derived EVs and to explore their potential implications for OA therapy.

Materials and methods: The miRNA cargo of EVs derived from ASCs obtained from different tissue sources and harvesting techniques was analyzed and compared. An extensive bioinformatics analysis was conducted to identify experimentally validated OA-related targets, pathways, and tissues affected by these miRNAs.

Results: All ASC-derived EVs contained miRNAs involved in cartilage homeostasis and immunomodulation. However, the EV-miRNA expression profiles differed depending on both the tissue origin and the harvesting method, leading to distinct molecular signatures. Bioinformatics predictions suggested that ASC-EVs derived from abdominal adipose tissue collected via surgical excision had a higher potential for cartilage protection and delaying OA progression, whereas ASC-EVs obtained from abdominal lipoaspiration were associated with enhanced anti-inflammatory properties.

Conclusions: These findings highlight the importance of considering both the anatomical origin and the harvesting method when aiming to optimize the therapeutic efficacy of ASC-EVs for OA treatment. This study provides a foundation for further functional and clinical research to evaluate the effectiveness of specific ASC-EVs in managing OA.

## 41. Feasibility study on the differentiation of mesenchymal stromal cells into fibroblasts for skin regenerative medicine applications

Federica Trenta^1^, Rosalba Monica Ferraro^2^, Elisa Borsani^3^, L. Assoni^1^, Camillo Almici^4^, Andrea Bianchetti^4^, Alessia Cavalleri^1^, Silvia Mutti^1^, Alessandro Leoni^1^, Luca Garufio^1^, Besjana Xhahysa^1^, Simona Bernardi^1^, S. Giliani^2^, Domenico Russo^1^, Federica Re^1^

^1^Department of Clinical and Experimental Sciences, University of Brescia, Unit of Blood Diseases and Bone Marrow Transplant, ASST Spedali Civili, Brescia, Italy. ^2^Department of Molecular and Translational Medicine, Institute of Molecular Medicine “Angelo Nocivelli", University of Brescia, Brescia, Italy. ^3^Department of Clinical and Experimental Sciences, Division of Anatomy and Physiopathology, University of Brescia, Brescia, Italy. ^4^Department of Transfusion Medicine, Laboratory for Stem Cells Manipulation and Cryopreservation, Blood Bank, ASST Spedali Civili, Brescia, Italy.

Objective: The primary goal of this study is to optimize and standardize a protocol for differentiating human mesenchymal stromal cells (hMSCs) into fibroblasts, which are the main cellular components of the skin dermis. Additionally, we evaluated the contribution of fetal bovine serum (FBS) and human platelet lysate (HPL) - recently introduced as a promising substitute - to the differentiation process.

Materials and methods: Umbilical cord-derived hMSCs (UC-hMSCs) were seeded at a density of 4,000 cells/ cm² and cultured in a fibroblastic medium (FM) consisting of DMEM supplemented with 100 ng/mL connective tissue growth factor and 50 μg/mL ascorbic acid. The medium was further supplemented with either 10% FBS or 5% HPL. As a control (CTR), cells were cultured in a complete medium without differentiation factors. Morphological, molecular, and protein analyses were performed on days 7, 10, and 14. Specifically, COLI gene expression was assessed using digital PCR, while the expression of Vimentin, CD44, αSMA, and COL11A1 was evaluated using real-time PCR. Additionally, immunohistochemical analysis for COLI and immunofluorescence analyses for Vimentin and CD44 were conducted.

Results: UC-hMSCs treated with FM+FBS showed higher COLI protein expression at all time points compared to FM without FBS and the CTR group. Given that growth factors may enhance differentiation, COLI molecular expression was compared between cells cultured with FBS and those with HPL. The results showed an increase, particularly in CTR+HPL samples compared to cells treated with FM alone. Furthermore, there was a statistically significant upregulation of Vimentin and *CD44* genes and a downregulation of *α-SMA* and *COL11A1* genes in UC-hMSCs cultured in FM, especially in the presence of HPL, at all time points. Consistently, immunofluorescence analysis revealed higher protein expression of Vimentin and CD44 in cells treated with FM supplemented with either FBS or HPL compared to the CTR group.

Conclusions: This study demonstrated the potential of UC-hMSCs to differentiate into fibroblasts when cultured with specific differentiation factors, especially with the addition of hPL. Further investigation is needed to elucidate the underlying pathways and molecules involved, to explore the feasibility of keratinocyte differentiation, and to apply these protocols to hUC-MSCs seeded into bioengineered scaffolds. Ultimately, this could lead to the development of 3D constructs capable of facilitating the wound healing process.

## 42. Use of the combination of microfat and platelet-rich plasma in a dog with an open fracture and tissue loss

Gian Luigi Vannucci^1^, Priscilla Berni^2^, Stefano Grolli^2^, Maurizio Del Bue^3^

^1^Veterinary Clinic Costa d’Argento - VetPartners Italia, Albinia, Grosseto, Italy. ^2^Department of Veterinary Science, University of Parma, Parma, Italy. ^3^Veterinary practitioner, Parma, Italy.

Objective: Open, comminuted, infected fractures with significant tissue loss in dogs are challenging to manage, often resulting in pseudoarthrosis and sometimes necessitating limb amputation. This case report describes a dog that sustained severe trauma from another dog’s bite. Microfragmented fat (microfat) has been proposed as a potential pro-regenerative support for soft and bone tissue repair. In this case, we evaluated the feasibility and efficacy of combining microfat and platelet-rich plasma (PRP) in a lesion that showed no tendency to heal due to infection and substantial tissue loss.

Materials and methods: A dog presented with an open radio-ulnar fracture with bone loss following dog bites, initially treated with external skeletal fixation. After 4 weeks, no bone healing was observed. Internal fixation using double plates (ulnar and radial) and a cancellous bone graft was then performed. Although initial bone growth was seen at 2 weeks, bone resorption was noted after an additional 2 weeks. Rather than opting for revision surgery, a conservative approach was chosen, using a combination of autologous microfat (prepared with the Tissue-Grinder, Hydra srl, Mirandola) and PRP. Adipose tissue was harvested from the falciform ligament, aseptically microfragmented, and mixed with 2 mL of autologous PRP. Under ultrasound guidance, a percutaneous injection was performed within minutes using a 20G needle, during the same surgical procedure, without a skin incision. The dog was discharged on the same day, and only one treatment session was administered.

Results: Follow-up visits at two-week intervals revealed progressive bone growth starting at the first evaluation. After 12 weeks, bone regeneration and union of the bone fragments were confirmed, with no further complications. At 12 months, the ulna plate was removed, and the dog regained its previous quality of life. The radial plate was left in place. Two-year follow-up confirmed complete bone consolidation with no signs of resorption.

Conclusions: This case report supports the hypothesis that combining microfat and PRP can be effective in promoting bone healing in dogs with significant tissue loss. The treatment can be delivered in a single, minimally invasive session, thereby avoiding additional surgical trauma to the tissues.

## 43. Mesenchymal stromal cell secretome and its potential in the treatment of polycystic ovary syndrome

Alessia Ventura, Maria Assunta Ucci

Department of Biomedicine and Prevention, University of Rome Tor Vergata, Rome, Italy.

Objective: Polycystic ovary syndrome (PCOS) affects approximately 10% of women of reproductive age worldwide. This study investigates whether conditioned medium (CM) derived from human mesenchymal stem cells (MSCs) isolated from discarded tissues, specifically adipose tissue (ADSC) and dental pulp (DPSC), can alleviate the main symptoms of PCOS in a mouse model. The ultimate goal is to identify specific molecules, particularly miRNAs and soluble factors, responsible for the therapeutic effects and to compare the efficacy of CM from these two tissue sources in treating PCOS.

Materials and methods: In this study, young female mice were first treated intravenously with the steroid hormone dehydroepiandrosterone (DHEA) for 28 consecutive days to induce PCOS-like symptoms. Thereafter, CMs were administered intravenously every other day for two weeks. Total RNA was extracted from the CMs for miRNA analysis, followed by sequencing on the Illumina platform. Differentially expressed miRNAs were identified and subsequently validated using real-time qPCR.

Results: Histological and molecular analyses confirmed that DHEA effectively induced a PCOS phenotype in mice, characterized by polycystic ovarian morphology, corpora lutea formation, and activated folliculogenesis. This was evidenced by a reduction in the primordial follicle pool and an increase in growing follicles compared to healthy controls. Therapeutic evaluation revealed that both CM treatments, particularly DPSC-CM, improved various parameters, including body weight, estrous cyclicity, ovarian structure, follicular activation, insulin sensitivity, and serum AMH levels. Notably, DPSC-CM alleviated the PCOS phenotype by reducing the number of cystic follicles and corpora lutea while preserving the follicular reserve. Finally, bioinformatics analysis of the miRNome, together with qPCR validation of miRNAs enriched in the MSC secretomes, confirmed the expected expression trends between CMs derived from different tissues.

Conclusions: These findings suggest that DPSC-CM contains factors that are more effective than ADSC-CM in mitigating the pathological changes associated with PCOS. Further analysis aims to identify specific miRNAs that may contribute to restoring ovarian function, particularly by preserving the primordial follicle pool.

## 44. Umbilical cord-derived mesenchymal stromal cells and hyaluronic acid-based biomaterials: optimizing cell delivery for enhanced therapeutic persistence

Laura Zocca^1^, Daniela Catanzaro^1^, Martina Bernardi^1^, Elena Merotto^2,3^, Luisa Galla^1^, Anna Merlo^1^, Francesca Elice^1^, Elisa Zolpi^4^, Cosimo Bleve^4^, Salvatore Fabio Chiarenza^4^, Giuseppe Astori^1^, Martina Piccoli^1,2^

^1^Advanced Cellular Therapy Lab, Haematology Unit, San Bortolo Hospital, Vicenza, Italy. ^2^Tissue Engineering Lab, Istituto di Ricerca Pediatrica Città della Speranza, Padova, Italy. ^3^Department of Industrial Engineering, University of Padova, Padova, Italy. ^4^Paediatric Surgery Unit, San Bortolo Hospital, Vicenza, Italy.

Objective: Mesenchymal stromal cells (MSCs) possess potent immunomodulatory and pro-regenerative properties, making them ideal candidates for regenerative medicine. However, in clinical practice, the importance of the injection medium is often overlooked, despite its crucial role in enhancing MSC homing, protecting cells from inflammatory environments, and ensuring their persistence at the target site. This study aimed to identify an optimized cell delivery strategy by combining umbilical cord-derived MSCs (UC-MSCs) with biomaterials that support cell retention and survival.

Materials and methods: We evaluated four clinically approved biomaterials for their potential use as cell carriers, considering key clinical requirements such as compatibility with endoscopic delivery in anatomically complex regions, ability to pass through fine-gauge needles, prolonged cell viability, and sustained secretion of regenerative factors in a three-dimensional environment. The effects of these biomaterials on MSC persistence, protection against inflammatory conditions, and functional activity were assessed *in vitro*.

Results: Among the tested biomaterials, two hyaluronic acid (HA)-based formulations, originally developed for osteoarticular treatments and dermal filler applications, demonstrated superior properties in terms of injectability, ease of handling, and cell viability. UC-MSCs cultured within these HA-based materials for up to seven days maintained high survival rates, exhibited robust proliferation, and showed enhanced secretion of key immunomodulatory and pro-regenerative cytokines, closely approximating their behavior in traditional 2D cultures. Notably, these biomaterials provided a protective microenvironment, mitigating potential damage from inflammatory conditions and improving MSC persistence at the site of administration.

Conclusions: These findings highlight the critical yet often neglected role of the injection medium in MSC-based therapies. HA-based biomaterials not only serve as effective delivery vehicles but also enhance MSC survival, homing, and functional persistence in hostile microenvironments.

## DECLARATIONS

### Availability of data and materials

Raw data are available at: https://osf.io/sy2b4/?view_only=db31a60c30e048a883069e8fcf798039

### Copyright

© The Author(s) 2025.

